# The impact of insole wedge height on knee alignment, joint space width, and patient-reported outcomes in medial knee OA

**DOI:** 10.1371/journal.pone.0354335

**Published:** 2026-08-25

**Authors:** Bashar Al Qaroot, Mohammad Sobuh, Mohammad Hamdan, Jihad Al Ajlouni, Farah Al-Imyan, Eman Al-Laham, Ibrahim Altubasi, Aws Khanfar

**Affiliations:** 1 Prosthetics and Orthotics department, The University of Jordan, Amman, Jordan; 2 Certified Prosthetist and Orthotist, Amman, Jordan; 3 School of Health and welfare, Jönköping University, Jönköping, Sweden; 4 Special surgery department, The University of Jordan, Amman, Jordan; 5 Consultant Orthopaedic Surgeon, Amman, Jordan; 6 Physical therapy department, The University of Jordan, Amman, Jordan; Somaiya Vidyavihar University K J Somaiya College of Engineering, INDIA

## Abstract

**Introduction:**

Lateral wedge insoles (LWIs) are a recommended conservative treatment for medial knee osteoarthritis (OA), yet the optimal wedge height remains unclear. This randomized controlled trial evaluated the effects of different LWI heights on radiographic outcomes in patients with medial knee OA.

**Methods:**

This study employed a randomized, controlled, parallel-arm design with a sequential, within-subject dose-escalation intervention arm. Thirty-four participants with mild-to-moderate medial knee OA (Kellgren- Lawrence grade <3) were randomized into a case group (n = 17) receiving custom LWIs (4 mm, 8 mm,12 mm, 16 mm) and a control group (n = 17) receiving standard care. Radiographic assessments (femorotibial angle [FTA], medial joint space width [MJSW]) and patient-reported outcomes (WOMAC) were performed at baseline and follow-up. Mixed ANOVA analyzed group and time effects.

**Results:**

The 12 mm LWI significantly reduced varus alignment (FTA: 177.3° ± 2.8 vs. 178.1° ± 2.7 with 4 mm, p < 0.05) in the non-dominant limb. No significant FTA change was observed in the dominant limb, suggesting that limb-specific loading history may modulate biomechanical responsiveness to orthotic correction. No significant FTA changes occurred in the dominant limb. MJSW remained stable in the case group but narrowed in controls (3.04 ± 0.7 mm vs. 3.16 ± 0.8 mm, p < 0.05). The 16 mm LWI was discontinued due to poor tolerability (11/17 participants). The LWI group also showed significant improvements in WOMAC total and pain scores compared to controls.

**Conclusion:**

Among the clinically tolerable LWI heights tested, the 12 mm wedge provided the best balance between biomechanical efficacy and patient tolerability. It significantly improved varus alignment (in the non-dominant limb), halted joint space narrowing, and reduced pain and overall symptoms. The 16 mm wedge was poorly tolerated (11/17 participants) and was therefore deemed clinically impractical. Future studies should explore personalized height selection and long-term outcomes.

## Introduction

Osteoarthritis (OA), a leading cause of pain and functional disability, is the most common musculoskeletal condition worldwide [1]. It affects weight-bearing joints, particularly the knee joint. The prevalence of knee OA increases with age, with females being more susceptible. In particular, ~ 13% of women and ~10% of men who are over 60 years old have symptomatic knee OA [2,3]. This is expected to further increase especially in developing countries where access to surgical intervention might not be readily available [4,5].

The etiology of knee OA remains unknown [6,7]. However, an interaction between systemic and biomechanical factors can contribute to its development and progression [3,8]. These factors include, but not limited to, obesity, previous knee injury or surgery, genetic susceptibility and knee malalignment [3]. In general, knee OA could develop in one or more of the three different knee compartments; medial tibiofemoral, lateral tibiofemoral and/or patellofemoral [9], of which, the medial tibiofemoral compartment is the most common [2,10]. This is due to the uneven load distribution during normal gait, as most of the total body’s load (60–80%) is transmitted through the medial side of the knee joint [2].

This uneven load distribution results in medially acting ground reaction force at the knee joint during the stance phase of gait. As a result, external knee adduction moment is generated. Thus, in relation to the femur, the tibia will rotate medially into a varus position [11]. This position is believed to be responsible for osteoarthritic changes in the medial knee compartment [12].

These changes are characterized by damaging the entire joint structure in an irreversible manner [2]. Including, articular cartilage degeneration, articular capsule stretching, synovitis, subchondral bone sclerosis, periarticular muscles weakness and ligaments laxity [9–11]. Consequently, restrictions of patients’ ability to perform daily activities due to knee pain, stiffness, joint effusion and limitation of knee range of motion [1,12,13].

Recommended treatment for medial knee OA varies depending on the severity of OA and its effects on the patient [13,14]. Severe cases may require an operative treatment. Whereas, mild to moderate cases could be managed with one or more of the non-operative treatment modalities, including activity modification, physical therapy, pharmaceutical (drugs or injections) and joint unloading therapy through orthotic intervention [14–16]. The aims of the non-operative treatment are predominantly to relieve the pain, improve the function and inhibit disease progression [14].

Joint unloading therapy by a lateral wedge insole (LWI) is one of the first-line modalities in the non-operative treatment of symptomatic knee OA [4]. It is a simple, safe and low-cost option that has been recommended in 12 of the 13 existing guidelines for the management of medial knee OA [17].

The goal of using a LWI is to increase foot pronation in order to centrally-realign the position of the ground reaction force vector relative to the knee joint during weight-bearing activities. This would reduce the loading at the medial knee compartment and consequently would mitigate knee pain and possibly cease OA progression [13,14].

Some published investigations indicated that the higher the LWI, the better the knee OA control [13,18]. However, the appropriate LWI height has, to the best of our knowledge, been never investigated. Therefore, the aim of this randomized control trial (RCT) is to investigate LWI effectiveness in knee OA management and to identify the best height for knee OA management based on radiographic evaluation.

## Method

The study was ethically approved by the institutional committee for research review at the University of Jordan hospital, Amman, Jordan (208/2020).

**Fig 1:**
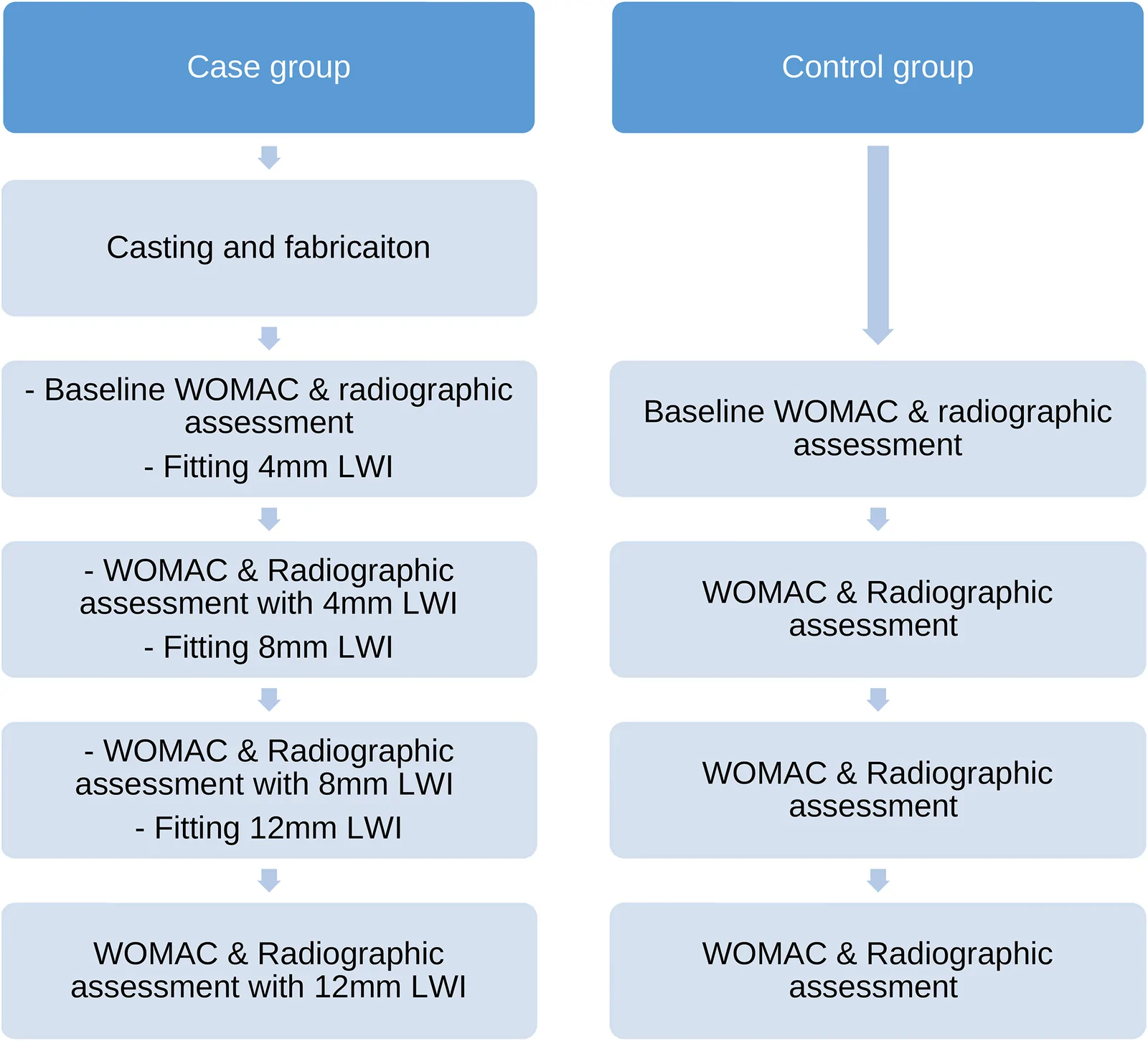
Overview of the flow of the investigation. LWI: lateral wedge insole. WOMAC: Western Ontario and McMaster Universities osteoarthritis index. The intervals between successive assessments was 4 weeks. In the case group, the interval between casting and delivering the 4 mm LWI was one week.

This study employed a combined design to address both the natural history of medial knee OA and the dose-response effects of LWIs. It consisted of two randomly assigned groups (control and case) with a repeated-measures structure. Participants in the control group were assessed at baseline and then at four consecutive follow-up visits (at 4-week intervals) without any orthotic intervention, to track disease progression. Participants in the case group were assessed at baseline and then after sequential 4-week periods of wearing 4 mm, 8 mm, 12 mm, and 16 mm LWIs. Thus, the ‘time’ factor in the analysis represents chronological follow-up for the control group and incremental intervention dose for the case group (see Fig 1).

### Participant selection

Participant recruitment took place at the University of Jordan Hospital’s orthopedic clinic. While all participants were recruited between September 1, 2020, and August 11, 2021, follow-up efforts continued until the final visit was completed on March 1, 2024. The extended timeline resulted from a concerted effort to retain and complete the protocol with a single non-adherent participant. Because enrollment was confined to the original one-year approval period, no ethical extension was needed. Eligibility required a clinical and radiographic diagnosis of bilateral mild knee osteoarthritis according to the American College of Rheumatology clinical criteria, with Kellgren-Lawrence grade < 3 [19]. Additionally, participants had to report persistent walking pain exceeding 3/10 on a numerical rating scale. Severe cases were excluded based on evidence suggesting that LWI demonstrate optimal efficacy in mild/moderate stages, ensuring the study population represented those most likely to benefit from LWI intervention [20]. Exclusion criteria included previous lower limb fracture or trauma, ankle or foot arthrodesis, musculoskeletal problems, currently using an orthosis or an ambulation aid to walk. Participants who fell within inclusion criteria were given a detailed participant information sheet. Potential participants who showed willingness to participate in the study contacted the chef investigator and were then asked to sign an informed consent form. Limb dominance was determined for each participant at the time of enrollment through self-report, by asking which leg they would favorably use to kick a ball. Table 1 shows participant’s information.

**Table 1:** Subject’s characteristics information. Where applicable, data presented as mean ± standard deviation.

Characteristic	Control Group (n = 17)	Case Group (n = 17)	p-value
Age, years	60.9 ± 9.8	59.9 ± 9.5	0.749
Body Mass Index (kg/m²)	29.8 ± 4.2	31.9 ± 5.6	0.207
Weight (kg)	78.2 ± 11.7	85.4 ± 20.9	0.204
Height (m)	162.1 ± 4.2	162.9 ± 11.2	0.784
Gender, n			0.500
Male	3	4	
Female	14	13	
Dominant Side, n			1.000
Right (R)	17	16	
Left (L)	0	1	

### LWI

**Fig 2:**
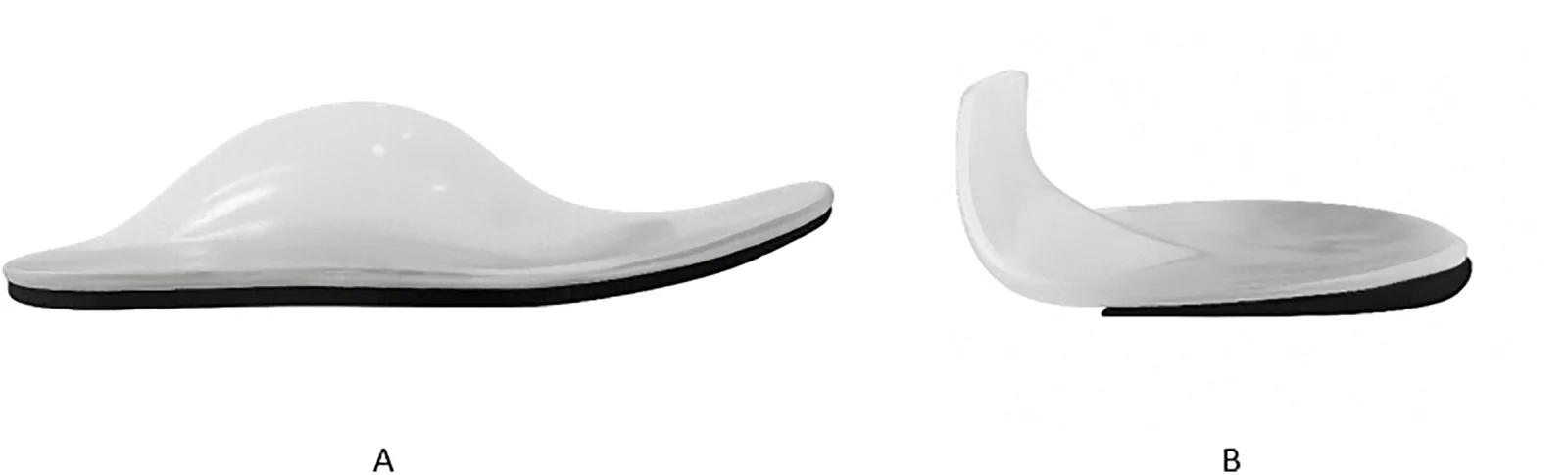
The lateral wedge insole. A) lateral view and B) frontal view. The insole in white and the lateral wedge in black.

The LWI were custom-made from a cast impression of each participant’s feet in the case group. The LWI was made of two glued parts (the insole and the lateral wedge). The insole covered the full length of the foot with medial arch support and was made from 2 mm Thermolyn Trolen (PE-LD). The lateral wedge was made from a black Neoprene rubber with different thicknesses (i.e., 4 mm, 8 mm, 12 mm and 16 mm thickness, as measured from the lateral side). The LWI was tapered laterally to align with the level of the medial side of the foot. Each lateral wedge thickness was glued to the inferior surface of the insole, and after each assessment, was unglued and changed with another thickness (Fig 2).

### Radiographic imaging

To assess knee OA in both control and case groups, short AP weight-bearing X-ray radiographs of both knees (knees extended and heels together) were acquired. Short, weight-bearing knee radiographs were selected as they are a validated and commonly used modality for assessing femorotibial alignment and medial joint space width in conservative knee OA trials, while minimizing radiation exposure and reflecting routine clinical practice [21,22]. All images were acquired with an X-ray beam (63 KV, 6.3 mAs), a focus of 0.5 mm and a focus to film distance of 115 cm. These images were used to measure the femorotibial angle (FTA) and the medial joint space width (MJSW) using the synapse software system (Version 4.4, Fujifilm, Japan).

**Fig 3:**
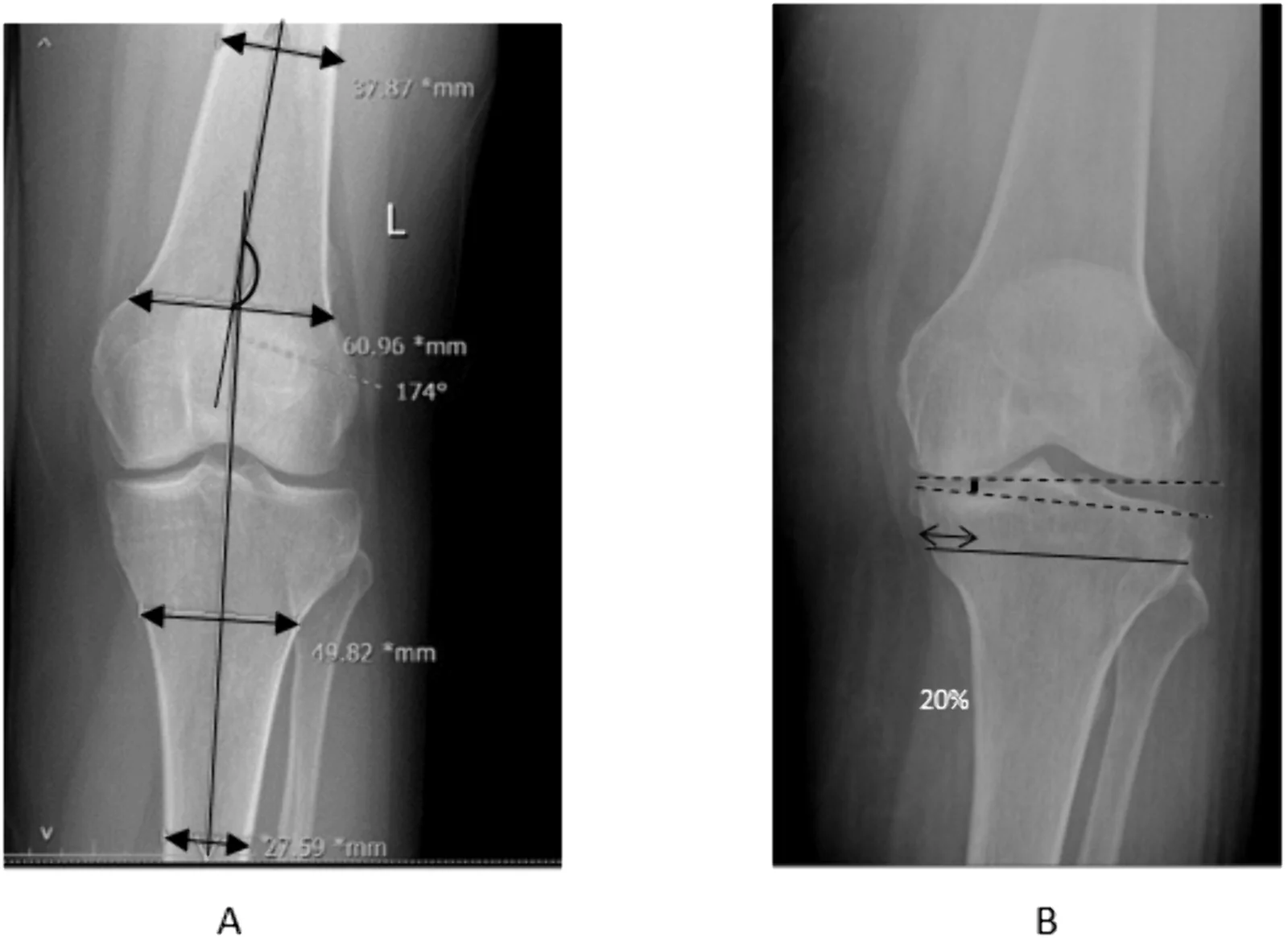
Radiographic Knee Metrics. **A.** Measurement of the femorotibial angle (FTA): The double arrows indicate the proximal and distal widths of the femoral and tibial shafts, respectively. Midpoints of these widths were connected to define the anatomical axes of each bone. The angle formed at the intersection of these axes represents the FTA. **B.** Measurement of the medial joint space width (MJSW): Dashed lines represent tangents to the femoral condyles and tibial plateaus. The solid horizontal line indicates the total width of the proximal tibia and was used to identify a point located 20% from the medial edge (marked by a double arrow). The MJSW was measured at this point as the perpendicular distance between the dashed lines, shown as a solid vertical line marked by an arrow.

The FTA was measured from the intersection of the femoral and the tibial anatomical axes. The femoral and tibial axes were drawn similarly by connecting the mid-points of the proximal and the distal respective shafts [21] (Fig 3.A). The resulting angle represents the relative alignment of the knee joint (measured from the lateral side), where a reduction in the FTA is considered as improvement in joint alignment in medial knee OA patients [21].

Regarding the MJSW, it was measured as the distance between two lines tangent to both the femoral condyles and the tibial plateaus at a point 20% from the medial tibial plateau (Fig 3.B) [22]. An increase in the distance is considered as improvement in joint width in medial knee OA patients.

### Patient reported outcomes

In addition to radiographic measures, patient-reported outcomes were assessed at each visit using the Western Ontario and McMaster Universities Osteoarthritis Index (WOMAC). The WOMAC is a validated, self-administered questionnaire that evaluates three domains: Pain (5 items), Stiffness (2 items), and Physical Function (17 items). Each item is scored on a Likert scale, with higher scores indicating worse pain, stiffness, and functional limitations. The total WOMAC score and individual sub-scores were calculated for each participant at every assessment time point.

### Assessment: Case group

Each participant in the case group was assessed five times: a baseline assessment without LWI and four other assessments while wearing LWIs of 4 mm, 8 mm, 12 mm, and 16 mm thickness, respectively. The interval between assessments was set at four weeks, which was considered sufficient to detect alterations in medial knee OA patients following LWI use [18,19,21–23]. This interval was also chosen to minimize the potential of participant dropout (due to the length of the study), ensure compliance, reduce the burden of long follow-up periods, and mitigate potential confounding factors such as adaptation effects or changes in symptoms unrelated to the intervention.

### Assessment: Control group

Each participant in the control group was assessed five times at four-week intervals, without LWI, to match the assessment schedule of the case group.

## Results

A total number of 44 bilateral medial knee OA participants were recruited. Of those, 10 participants withdrew from the study due to different reasons; including, coronavirus pandemic, discomfort while wearing the first given LWI, transportation difficulties and some of them dropped out of the study for no explained reasons.

The remaining 34 participants were randomly divided using a dynamic, sequential randomization approach where each participant’s group allocation was determined at the time of enrollment using a computerized random number generator. This real-time allocation strategy was developed to accommodate our study’s practical constraints; specifically, the inability to predict final enrollment numbers at the study’s onset. The system automatically maintained balanced group sizes throughout the recruitment period. Importantly, this approach reflected clinical situation, where participants present randomly, while still maintaining the scientific integrity of randomized assignment.

Based on randomization, 17 participants were allocated to the case group (59.9 ± 9.5 years old) who have received the LWIs and 17 participants were allocated to the control group (60.9 ± 9.8 years old) with no orthotic intervention. Participants in both groups were blinded about the presence of the other group. Of the 17 participants in the case group, 11 were unable to wear the 16 mm LWI due to pain and discomfort and were withdrawn from this condition. The remaining 6 participants completed the 16 mm condition. Due to the high dropout rate (64.7%), the 16 mm data were excluded from the primary analysis because the reduced sample size (n = 6) would have compromised statistical power and introduced significant selection bias. However, an exploratory analysis of the 6 completers is provided as supplementary material (see S1 File). Exploratory analysis of the 16 mm completers (n = 6) revealed a non-significant trend toward further FTA improvement compared to 12 mm (mean difference = −0.6°, p = 0.18). Due to the small sample size and potential selection bias, these findings should be interpreted with caution and are provided in supplementary material (S1 File).

### Statistical analysis

Mixed ANOVA was performed to analyze the effects of group and different LWI heights on WOMAC scores, FTA, and MJSW. In this model, the ‘time’ factor represents the chronological follow-up visits for the control group and the incremental wedge-height condition for the case group. Consequently, the group×time interaction was specifically interpreted as a test of whether the dose-response trajectory in the case group differed significantly from the natural progression trajectory in the control group.

Since a total number of 34 participants completed the study from the original 44 recruits, it should be noted that the final sample size may have limited the statistical power to detect smaller effect sizes, particularly in the side-specific analyses.

Baseline characteristics between the control and case groups were compared using independent samples t-tests for continuous variables and a Chi-square test/Fisher’s exact test for categorical variables (gender and side dominance, respectively)

### Post-hoc power analysis

Post-hoc power analyses were performed for the primary outcomes (FTA and MJSW) and WOMAC subscales based on the observed effect sizes (partial η²) from the Mixed ANOVA, with α = 0.05 and the achieved sample size (n = 34). As shown in Table 2, adequate power (0.82) was observed for the significant FTA interaction on the left side. However, power was low for the right side FTA (0.06), right side MJSW (0.15), and WOMAC stiffness (0.32) and physical function (0.42) subscales. Thus, null findings for these outcomes should be interpreted with caution.

**Table 2:** Post-hoc power analysis for primary and secondary outcomes based on observed effect sizes (partial η²) from Mixed ANOVA (α = 0.05, n = 34).

Outcome	Effect Size (partial η²)	Observed Power
FTA (left side) – group×time interaction	0.053	0.82
FTA (right side) – group×time interaction	0.001	0.06
MJSW (left side) – group×time interaction	0.032	0.61
MJSW (right side) – group×time interaction	0.009	0.15
WOMAC Stiffness – group×time interaction	0.015	0.32
WOMAC Physical Function – group×time interaction	0.020	0.42

### Patient reported outcomes (WOMAC)

**Fig 4:**
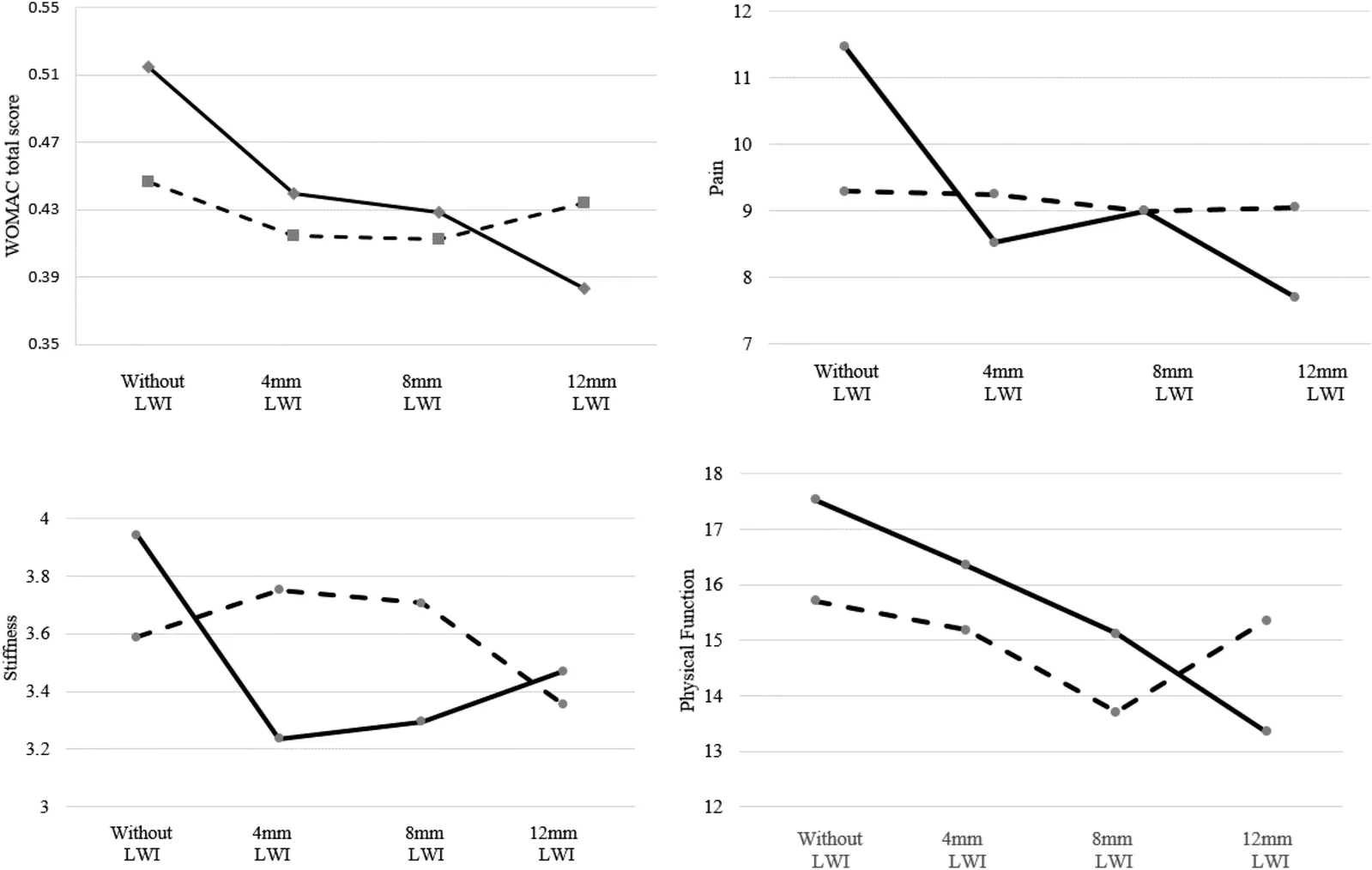
Changes in WOMAC total and sub scores. Changes in WOMAC total and stiffness, pain, and physical function sub-scores across conditions without and with 4 mm, 8 mm, and 12 mm lateral wedge insoles (LWI). The solid line represents the case group, while the dashed line represents the control group. The higher the score, the worse the condition is.

Regarding the total WOMAC score, Mauchly’s test of sphericity was not significant, therefore sphericity assumption was met. A significant interaction effect (group*time) was found (F (3,93) = 2.84, p = 0.042). Post-hoc analysis revealed that while the control group showed no significant change at any time point, the case group demonstrated a significant decline (improvement) in the total score. Specifically, the case group’s scores at the 3rd (8 mm LWI) and 4th (12 mm LWI) time points were significantly lower than at baseline (p = 0.030 and p = 0.002, respectively) (Fig 4).

Regarding WOMAC pain sub-score, Mauchly’s test of sphericity was not significant, therefore sphericity assumption was met. A significant interaction effect (group*time) was found (F (3,93) = 3.92, p = 0.011). The case group reported significant reductions in pain at the 2nd (4 mm LWI), 3rd (8 mm LWI), and 4th (12 mm LWI) visits compared to baseline (all p < 0.05). No significant changes were observed in the control group (Fig 4).

Regarding WOMAC stiffness and physical function sub-scores, Mauchly’s test of sphericity was significant, therefore sphericity assumption was not met, and Greenhouse-Geisser test was used. There were no significant interaction effects for the stiffness (p = 0.553) or physical function (p = 0.098) sub-scores. However, a significant main effect of time was observed for physical function (p = 0.03), with follow-up tests indicating a significant improvement in the case group at the 4th time point (12 mm LWI) compared to baseline (p = 0.045) (Fig 4).

The absence of significant interaction effects for the stiffness and physical function subscales should be interpreted cautiously, as post-hoc power analysis indicated insufficient power (observed power = 0.32 and 0.42, respectively) to detect small-to-moderate effect sizes. The significant main effect of time for physical function suggests a potential treatment benefit, but this finding requires confirmation in larger samples.

### FTA

On the right side, Mauchly’s test of sphericity was not significant, thus, sphericity assumption was met. The results indicate no significant interaction effect (group×time) on the FTA (F (3,90) = 0.027, p = 0.994). Also, no significant group nor time effects (F (1,30) = 0.055, p = 0.816) and (F (3,90) = 0.267 p = 0.849), respectively.

**Fig 5:**
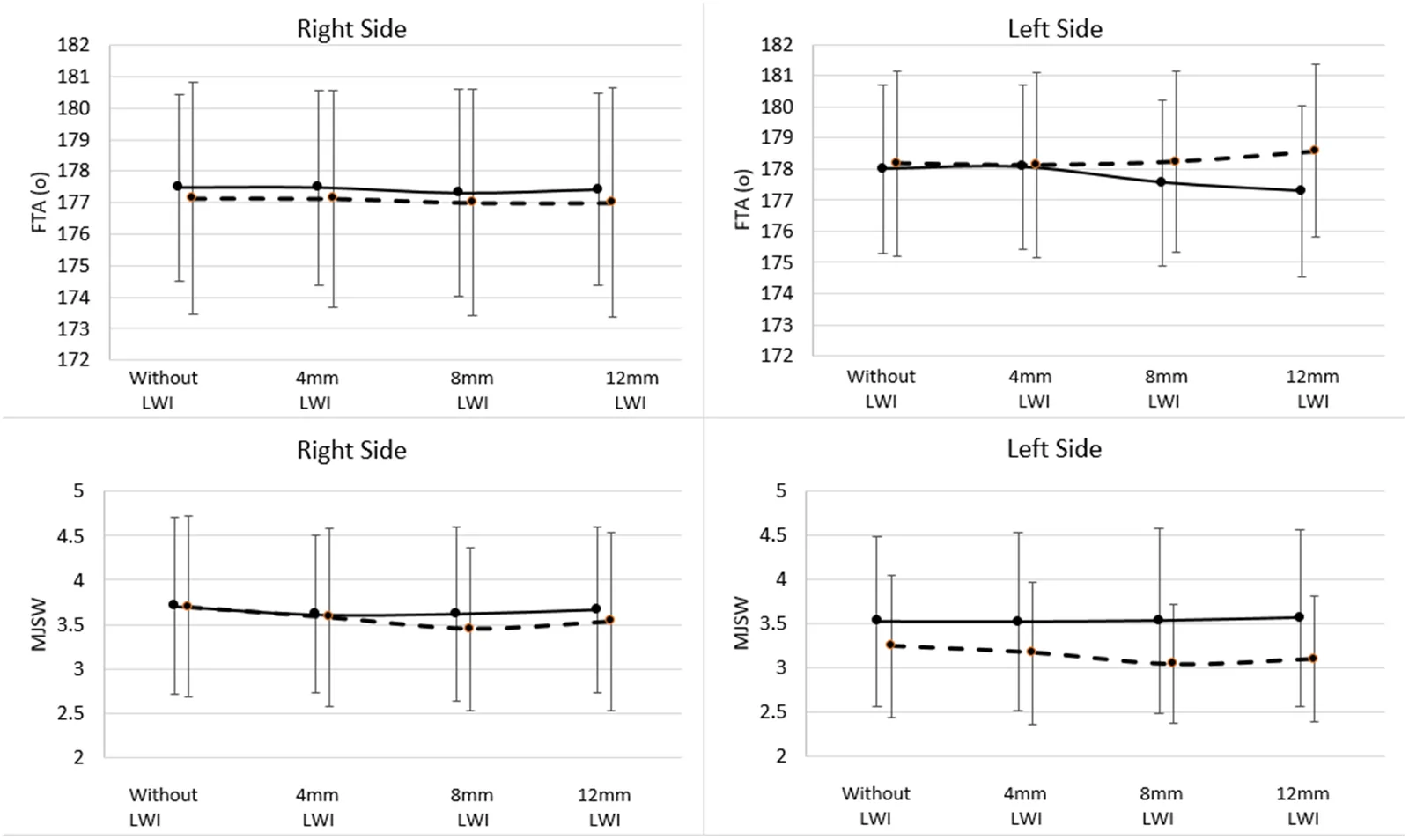
Knee Metrics Across LWI Conditions. Mean femorotibial angle (FTA) and medial joint space width (MJSW) of the right and left knees across conditions without and with 4 mm, 8 mm, and 12 mm lateral wedge insoles (LWI). Error bars represent standard deviations. The solid line is the case group and the dashed line is the control group. Notably, this significant FTA improvement was observed exclusively on the left side, which was the non-dominant limb in the majority (16/17) of participants. No significant FTA changes were detected on the right (dominant) side in either group.

On the left side, Mauchly’s test of sphericity was significant, thus, sphericity assumption was not met. Accordingly, Greenhouse-Giesser test was used for further analysis. The results indicate a significant interaction effect (group×time) (F (3,93) = 5.231, p = 0.005). Particularly, for the control group there was no significant change among different conditions. However, for the case group, there was a significant decrease with 12 mm LWI (177.3 ± 2.8) compared to the 4 mm LWI (178.1 ± 2.7) (Fig 5).

### MJSW

On the right side, Mauchly’s test of sphericity was significant, thus, sphericity assumption was not met. Accordingly, Greenhouse-Giesser test was used for further analysis. The results indicate no significant interaction effect (group*time) (F (3,90) = 0.883, p = 0.419). Also, there was no significant group nor time effects (F (1,30) <.001, p = 0.993) and (F (3,90) = 1.563 p = 0.204) respectively.

On the left side, Mauchly’s test of sphericity was not significant, thus, sphericity assumption was met. The results indicate a significant interaction effect (group*time) (F (3,93) = 3.036, p = 0.033). For the case group, there was no significant change among different time points. However, for the control group there was a significant decrease in the third visit (3.04 ± 0.7) compared to the first baseline visit (3.16 ± 0.8) (Fig 5).

## Discussion

The current investigation shows that MJSW reduced in the control group (both right and left sides) over successive visits where it was statistically significant in the 3rd visit for the left side (3.04 ± 0.7). This aligns with previous studies that reported deterioration and joint space narrowing, in knee OA, without intervention [24]. In contrast, MJSW remained unchanged in the case group, indicating that the LWI may have helped in preventing joint narrowing and thus decelerating joint degeneration [24]. However, the 4-week intervention period does not establish whether this structural preservation translates into sustained clinical benefit or delayed disease progression beyond the intervention period. Longer-term follow-up studies (e.g., 12 months) are needed to confirm the durability of these effects.

In accordance with previous studies that showed reduced varus alignment in knee OA patients wearing LWI [24–26], the current study revealed a reduction in varus knee angle as identified by the FTA whilst wearing 4 mm, 8 mm, and 12 mm LWIs. This reduction was statistically significant when a 12 mm LWI was worn on the left side (177.3 ± 2.8), suggesting that the higher the LWI, the more effective it is as long as it remains pain-free. Since the LWIs corrects knee alignment, it may reduce biomechanical stress on the medial knee compartment, potentially slowing the progression of OA [27]. Additionally, this realignment could lower the external knee adduction moment (EKAM) by redistributing joint forces more evenly [27].

While full-length lower-limb imaging provides comprehensive assessment of the mechanical axis, the present study was designed to evaluate knee-level structural changes relevant to conservative orthotic management rather than global limb deformity.

Both the slight increase in FTA and reduction in MJSW in the control group demonstrated progressive varus malalignment (Table 3) and OA deterioration compared to the case group, providing further evidence for the therapeutic value of LWI in managing medial knee OA progression.

**Table 3:** Femorotibial angle (FTA, degrees) and medial joint space width (MJSW, mm) measurements at baseline and follow-up visits for control and case groups (4 mm, 8 mm, and 12 mm LWIs). Values represent mean (SD) for right and left knees. LWI: lateral wedge insole.

Group	Visit / Intervention	Right Knee	Left Knee
**FTA**			
**Control**	1st Visit (Baseline)	177.12 ± 3.68	178.17 ± 2.96
	2nd Visit	177.11 ± 3.42	178.11 ± 2.97
	3rd Visit	177.00 ± 3.60	178.23 ± 2.90
	4th Visit	177.00 ± 3.64	178.58 ± 2.76
**Case**	Baseline (No LWI)	177.47 ± 2.96	178.00 ± 2.71
	4mm LWI	177.47 ± 3.08	178.00 ± 2.63
	8mm LWI	177.31 ± 3.28	177.56 ± 2.65
	12mm LWI	177.41 ± 3.04	177.29 ± 2.75
**MJSW**			
**Control**	1st Visit (Baseline)	3.70 ± 1.01	3.16 ± 0.79
	2nd Visit	3.57 ± 1.00	3.16 ± 0.80
	3rd Visit	3.44 ± 0.91	3.04 ± 0.66
	4th Visit	3.53 ± 1.00	3.09 ± 0.70
**Case**	Baseline (No LWI)	3.70 ± 0.99	3.52 ± 0.96
	4mm LWI	3.61 ± 0.88	3.51 ± 1.00
	8mm LWI	3.62 ± 0.98	3.53 ± 1.04
	12mm LWI	3.66 ± 0.93	3.56 ± 0.99

**Fig 6:**
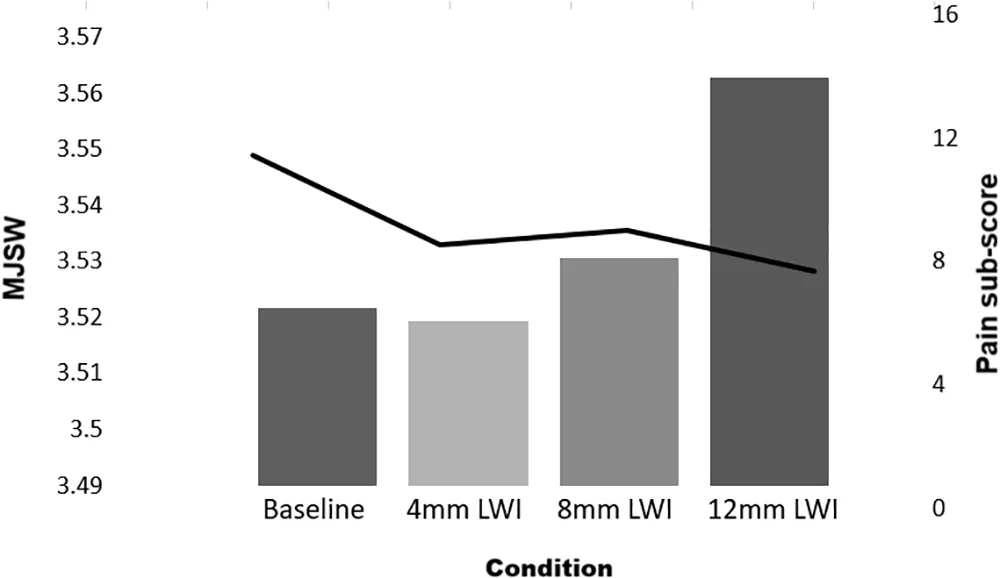
Visualizing the pathway from structural improvement to symptomatic benefit. The bars demonstrating the increase in medial joint space width (MJSW) from baseline to the 12 mm lateral wedge insole (LWI) condition in the case group (left knee), indicating structural improvement. Corresponding line graph showing the progressive reduction in WOMAC Pain scores across the intervention period, demonstrating concurrent symptomatic relief. Together, these panels form a clinical argumentation loop, linking objective biomechanical data to tangible patient benefit.

Current findings offer preliminary guidance for identifying an optimal LWI height to enhance treatment efficacy (i.e., 12 mm LWI). This structural improvement was paralleled by significant patient-reported benefits, as evidenced by the reduction in total WOMAC score and pain at the 12 mm LWI condition. The clinical significance of these findings is best appreciated by integrating the radiographic and patient-reported outcomes, as visualized in Fig 6. This Fig illustrates the direct relationship between the structural improvement in joint space and the concurrent reduction in patient-reported pain, closing the loop between laboratory data and tangible clinical benefit.

The 16 mm LWI could not be adequately tested due to poor tolerability, with 11 of 17 participants discontinuing this condition. While an exploratory analysis of the 6 completers suggested a possible trend toward greater biomechanical correction, the high dropout rate precludes any definitive conclusion about the efficacy of the 16 mm height. Critically, this high dropout rate is itself a clinically significant finding: it demonstrates that the 16 mm wedge, regardless of its theoretical biomechanical potential, is impractical for routine clinical use due to discomfort. This discomfort is possibly associated with asymmetric muscles activation and the body’s adaptive challenges to the more pronounced foot position adjustment [26,28]. This highlights a critical clinical consideration for LWI prescription in which the therapeutic height must balance biomechanical efficacy with wearability. While higher LWI may offer greater advantages, the optimal height represents a compromise between therapeutic benefit and practical tolerability, as clinical effectiveness ultimately depends on use. Thus, the 12 mm wedge represents the optimal balance between biomechanical efficacy and clinical feasibility among the heights tested.

FTA reduction was statistically significant only on the left (non-dominant) side. The majority of participants (16/17) were right-leg dominant. This side-specific effect could hypothetically reflect differential biomechanical responsiveness rather than a failure of the intervention. Limb dominance is associated with asymmetric loading history, with the dominant limb experiencing higher cumulative impact forces and propulsive demands during gait [29]. This chronic, elevated mechanical stress may lead to subtle but functionally significant adaptations in cartilage composition, subchondral bone stiffness, and periarticular soft tissues. These adaptations could render the dominant joint less responsive to acute biomechanical correction, inhibiting the kinematic chain effect of foot pronation before producing a measurable FTA shift within the 4-week intervention period.

Importantly, this interpretation is supported by the absence of side-specific differences in patient-reported outcomes (WOMAC) and MJSW preservation, which were observed bilaterally. Thus, the absent FTA effect on the dominant side does not indicate that the LWI is ineffective, but rather that limb dominance is a clinically relevant factor modulating biomechanical responsiveness. This finding does not undermine the overall therapeutic benefit of the 12 mm LWI, which is supported by convergent evidence from patient-reported outcomes and joint space preservation. Additionally, post-hoc power analysis revealed low statistical power (0.06) for this comparison, suggesting the null finding on the dominant side may also be partially attributable to insufficient sample size.

Lastly, while previous studies support using higher lateral wedge insole (LWI) heights for knee symptom relief [13,30–32], their methodology had key limitations. These studies tested only one wedge height per patient, making it impossible to determine if another height might work better for that individual. Results could reflect patient-specific factors rather than true treatment effects. Additionally, adjustments were permitted during trials [30], and inconsistent insole materials [30] may have biased outcomes. In contrast, our study directly compared multiple LWI heights on same participants under standardized conditions, eliminating these confounding factors. Furthermore, beyond symptomatic relief, our study provides preliminary evidence of potential disease-modifying effects. While most previous LWI trials focused on pain reduction or biomechanical parameters such as the external knee adduction moment (EKAM) [24,27,28], our radiographic findings demonstrate stabilization of MJSW in the case group compared to progressive narrowing in controls. This suggests that LWIs may decelerate joint space narrowing (a key structural marker of OA progression) within a relatively short intervention period. To our knowledge, this is among the first studies to demonstrate both dose-dependent biomechanical improvement and structural preservation concurrently, while also systematically documenting tolerability across multiple heights. This combination of findings provides a more comprehensive evidence base for clinical prescription practice.

### Limitation

This study has several limitations that should be considered when interpreting the results. First, the final sample size (n = 34), while sufficient to detect the primary effects, limited statistical power for side-specific and subgroup analyses. Post-hoc power analysis revealed low power for the dominant-side FTA (0.06) and WOMAC stiffness/physical function subscales (0.32–0.42); thus, these null findings may reflect insufficient power rather than a true absence of effect. Second, while baseline demographics were well-balanced, we did not systematically control for potential confounders such as disease duration, physical activity level, occupational demands, comorbidities, or medication dosage/frequency, all of which may influence disease progression and treatment response.

Third, the sequential administration of different wedge heights within the case group partially confounds the ‘time’ factor with intervention dose. Consequently, the group×time interaction compares the dose-response trajectory against natural progression, rather than a pure chronological treatment effect. Fourth, the FTA improvement was observed only in the non-dominant limb, limiting the generalizability of this structural finding; this may reflect differential loading history or insufficient power on the dominant side. Fifth, the 16 mm condition was excluded from primary analysis due to a 64.7% dropout rate, and the small number of completers (n = 6) precludes definitive conclusions about its efficacy. Thus, our conclusion regarding 12 mm efficacy is limited to the clinically tolerable range of heights tested.

Finally, the 4-week follow-up period, while sufficient to detect acute changes, does not establish the long-term sustainability of these effects. Extended follow-up studies are needed to determine whether the observed MJSW stabilization translates into delayed disease progression and sustained clinical benefit. Additionally, while WOMAC provides validated patient-reported outcomes, we did not include objective functional measures (e.g., gait analysis, timed walk tests), which should be incorporated in future studies. Assessment of global mechanical axis alignment using full-length lower-limb imaging was also beyond the scope of this study.

## Conclusion

This study provides valuable insights into the dose-response relationship of LWIs in managing medial knee OA. Among the clinically tolerable heights tested, the 12 mm LWI offered the best balance between biomechanical efficacy and patient tolerability, significantly improving varus alignment (in the non-dominant limb), halting joint space narrowing, and reducing pain and symptoms. The 16 mm LWI, while potentially more effective biomechanically, was poorly tolerated by the majority of participants and is therefore clinically impractical. These findings support the use of 12 mm LWIs as a conservative management strategy and highlight the need for personalized height selection based on individual tolerability.

The varus-correcting effect of the 12 mm LWI was observed exclusively in the non-dominant limb, suggesting that limb-specific loading history may modulate biomechanical responsiveness to orthotic correction. This observation does not undermine the overall therapeutic benefit of the intervention, as symptom improvement and joint space preservation were observed bilaterally. However, it highlights the complex interplay between joint loading, limb dominance, and OA progression—an area that warrants further investigation in future trials designed to account for limb dominance as a stratification variable.

This study contributes to the growing body of evidence supporting conservative management of knee OA, offering a simple and low cost strategy. Future research should explore long-term outcomes (e.g., 12-month follow-up) to determine whether the observed structural preservation translates into sustained clinical benefit and delayed disease progression. Additionally, studies investigating individual biomechanical factors, patient tolerance, long-term adherence, dynamic gait changes, and the potential for combining LWIs with other therapies are needed to optimize personalized treatment decision.

These findings should be interpreted within the context of knee-level assessment and short-term conservative management rather than whole-limb deformity correction.

## Supporting information

S1 File Supporting information file. (XLSX)
