# Supplementary material for: The impact of insole wedge height on knee alignment, joint space width, and patient-reported outcomes in medial knee OA
Source: PLoS One. 2026 Aug 25;21(8):e0354335. doi: 10.1371/journal.pone.0354335 (PMC13505888; doi:10.1371/journal.pone.0354335)
Supplement: Supplementary file 1 [file pone.0354335.s002.docx]

Starting from the phenomenon of “increased hip replacement rates in dominant limbs,” we can infer that “mechanical stress” may be the core influencing mechanism. Concurrently, confounding factors such as occupation and BMI cannot be overlooked. Ultimately, this leads to the specific application of the “LWI (lateral wedge insole) study,” forming a complete logical chain of “phenomenon - mechanism - application.” However, the current argument has several critical shortcomings that require refinement: 1. Unclear mechanism explanation: The specific pathway by which “increased mechanical stress on the dominant side compromises BMI effectiveness” remains undefined. The impact of mechanical stress on BMI's protective role could either directly negate its benefits or indirectly interfere through mediating factors like joint load distribution or local tissue metabolism. This key mechanism remains unclear and warrants further exploration. 2. Missing contextual basis: When mentioning that “LWI effects were not observed on the right side,” the paper fails to provide the widely accepted supporting rationale. Most individuals exhibit physiological characteristics of right-handedness/right-footedness, which may lead to higher frequency and greater load on the right limbs during daily activities. This could consequently affect the intervention efficacy of LWI. The omission of this contextual information may leave non-specialist readers with a somewhat unclear understanding of the “right-side specificity.”

Table Optimization Directions：

1. Table 1 Improvement: Adopt the three-line table format mandated by clinical research standards, clearly distinguishing headers, data columns, and footers. Additionally, incorporate gender and age as critical confounding factors during data analysis. Employ stratified analysis and multivariate regression to eliminate their interference, ensuring result accuracy.
2. Table 2 Improvement: Similarly, adhere to the three-line table format and supplement with tests or adjustments for baseline characteristic balance (e.g., gender, age). If other potential confounders exist (e.g., disease duration, comorbidities), incorporate them into the control scope to avoid compromising the reliability of conclusions. Enhancing the Clinical Argumentation Loop The logical framework of clinical research must form a complete loop: “Data Support → Clinical Validation → Patient Benefit.” “Clinical image comparisons” serve as the core bridge linking ‘data’ to “patient benefit.” The original text only addresses “multi-height data comparisons under standardized conditions,” achieving laboratory-level variable control but failing to provide critical clinical support through actual images:

1.Supplement with pre- and post-intervention joint imaging (e.g., X-ray, MRI) to visually demonstrate structural benefits like joint space widening and osteophyte reduction.

2. Incorporate functional assessment images (e.g., gait analysis diagrams, pain VAS trend charts) to demonstrate how data differences translate into tangible clinical outcomes like “reduced joint pain” and “enhanced walking ability.” Without such visual validation, the conclusion that “LWI studies show superiority” lacks the critical link between laboratory data and real patient benefits, failing to form a complete clinical evidence loop.
