# Supplementary material for: The impact of insole wedge height on knee alignment, joint space width, and patient-reported outcomes in medial knee OA
Source: PLoS One. 2026 Aug 25;21(8):e0354335. doi: 10.1371/journal.pone.0354335 (PMC13505888; doi:10.1371/journal.pone.0354335)
Supplement: Supplementary file 2 [file pone.0354335.s004.docx]

| Comment | Response |
| --- | --- |
| Reviewer 1 | |
| Why were patient-reported outcomes (PROs) not included? | We thank the reviewer for this critical and insightful comment. We completely agree that patient-reported outcomes (PROs) are essential for evaluating the clinical relevance of interventions for knee OA.  In our initial submission, we focused exclusively on the radiographic outcomes (FTA and MJSW) to maintain a clear and concise narrative on the structural and biomechanical effects of different LWI heights. However, we did indeed collect PROs throughout the study using the Western Ontario and McMaster Universities Osteoarthritis Index (WOMAC).  In direct response to the reviewer's comment, we have now integrated the full WOMAC results into the manuscript. The data show significant and clinically meaningful improvements in the case group compared to the control group, particularly for the Total WOMAC score and the Pain sub-scale. These findings provide compelling evidence for the symptomatic benefit of lateral wedge insoles, directly addressing the question of clinical relevance.  The following sections have been updated:   - **Methods:** Added a description of the WOMAC data collection. - **Results:** Added a new subsection detailing the WOMAC results. - **Discussion:** Integrated the interpretation of the WOMAC findings with the radiographic results. - **Abstract and Conclusion:** Updated to reflect the new clinical findings.   We believe the inclusion of this data substantially strengthens our paper and we are grateful for the reviewer's guidance on this matter |
| **Is the study sufficiently powered?** | **We acknowledge the reviewer's concern regarding statistical power. The final sample size was indeed smaller than initially planned due to participant dropout, a common challenge in longitudinal studies. A post-hoc power analysis confirmed that the study was underpowered to detect smaller effect sizes, particularly for the inter-limb differences observed. Therefore, the significant findings on the left limb should be interpreted with caution as preliminary evidence. We have revised the manuscript to explicitly state this limitation and to frame our conclusions as generating hypotheses for future research rather than providing definitive clinical guidance. We believe the novel within-subject, multiple-height design provides valuable data that can be used to inform sample size calculations for larger, definitive trials.**  **In the results:** Since a total number of 34 participants completed the study from the original 44 recruits, it should be noted that the final sample size may have limited the statistical power to detect smaller effect sizes, particularly in the side-specific analyses.  In limitation: this was addressed. |
| **The post-hoc explanation for limb dominance is speculative** | We thank the reviewer for this comment, which allows us to clarify an important point. Limb dominance was indeed assessed during the study. As previously stated in the Discussion: **"In the current study (where the right leg was predominantly the dominant side)..."**  This statement was based on data collected from participants regarding their leg dominance (through direct questioning about their kicking leg or leg used to step up on a stair). We apologize that this methodological detail was not explicitly described in the Methods section, creating the impression that it was a purely post-hoc consideration.  In response to the reviewer's feedback, we have now:   1. **Added a sentence to the Methods section** under 'Participant selection' to clearly state that limb dominance was recorded (added to table 1 as well). 2. **Refined the language in the Discussion** to more carefully frame our interpretation of these results as a possible hypothesis derived from the observed dominance data, acknowledging that other unmeasured factors could also contribute to the differences.   We believe these revisions provide greater methodological transparency and strengthen the interpretation of our findings |
| Reviewer 2 | |
| **Points 1 & 2 (Unclear Mechanism & Missing Context)** | We sincerely thank the reviewer for this exceptionally insightful critique that pushes us to refine the theoretical framework of our findings. We agree that the logical chain from phenomenon to mechanism to application needed greater clarity and depth. We have now substantially revised the Discussion to address this by:  **1. Providing the Missing Contextual Basis:**  We have amended the text to establish the foundational, well-accepted context that was previously implied (discussion is amended now and highlighted).  **2. Defining the Specific Mechanistic Pathway:**  The reviewer correctly noted that the pathway from "mechanical stress" to "compromised LWI effectiveness" was vague. We have now elaborated on a reasonable, step-by-step biomechanical and structural pathway:   - **Step 1 (Chronic Stress):** The dominant limb is subject to greater cumulative mechanical stress over a lifetime. - **Step 2 (Structural Adaptation):** This chronic stress can accelerate joint degeneration and lead to more established structural adaptations in medial knee OA (e.g., greater cartilage wear, bone remodeling, ligamentous laxity). We link this to the cited literature on dominant limb pathology [29]. - **Step 3 (Biomechanical Stiffness):** These structural changes may result in a biomechanically "stiffer" or less responsive joint. The joint's alignment may be more fixed, and the soft tissues less capable of facilitating the postural adjustments induced by the LWI. - **Step 4 (Impact on LWI Mechanism):** The primary mechanism of a LWI is to cause subtle foot pronation, thereby shifting the ground reaction force and reducing the external knee adduction moment (EKAM). We hypothesize that a more degenerated and structurally adapted dominant limb has a **diminished capacity to translate the LWI's foot-level correction into a meaningful knee-level realignment.** The higher baseline stress and altered joint mechanics may effectively "overwhelm" the intervention's biomechanical effect.   This elaborated pathway moves from a general observation to a specific hypothesis about why our radiographic outcomes (FTA, MJSW) showed a weaker response on the dominant side. |
| Table Optimization Directions 1. Table 1 Improvement: Adopt the three-line table format mandated by clinical research standards, clearly distinguishing headers, data columns, and footers. Additionally, incorporate gender and age as critical confounding factors during data analysis. Employ stratified analysis and multivariate regression to eliminate their interference, ensuring result accuracy. | We thank the reviewer for this critical suggestion to enhance the clarity and statistical thoroughness of our baseline data presentation. We have fully implemented both recommendations as follows:   1. **Three-Line Table Format:** Table 1 has been reformatted into the mandated three-line table format, removing all vertical and unnecessary horizontal lines for a cleaner and more professional presentation. 2. **Also, as** requested, we have now formally compared the baseline characteristics of the control and case groups. We have added a new column to Table 1 displaying the p-values from independent samples t-tests (for age, BMI, weight, height) and tests for gender and dominince. The results confirm that there were no statistically significant differences between the groups for any of these variables at baseline (p > 0.05).   Regarding the use of stratified analysis or multivariate regression to control for these factors in the outcome analysis: our primary statistical test, the Mixed ANOVA, is a robust model that inherently accounts for within-subject variability and compares the change over time between groups. Given that the groups were demonstrably equivalent at baseline (as now proven in the revised Table 1), the significant interaction effects (group*time) we report are unlikely to be driven by confounding from age or gender. However, we acknowledge the reviewer's point and have added a sentence to the statistical analysis section and the limitations to reflect this consideration. |
| 2. Table 2 Improvement: Similarly, adhere to the three-line table format and supplement with tests or adjustments for baseline characteristic balance (e.g., gender, age). If other potential confounders exist (e.g., disease duration, comorbidities), incorporate them into the control scope to avoid compromising the reliability of conclusions. | We thank the reviewer for this suggestion to enhance the clarity and strength of our results table.   1. **Three-Line Table Format:** We have reformatted Table 2 into the required three-line table format for improved clarity and presentation. 2. 3. **Addressing Baseline Balance and Confounders:** We agree that ensuring results are not confounded by baseline differences is paramount. We would like to clarify the properties of our chosen statistical model, the Mixed ANOVA:    - This model is specifically designed for longitudinal data and **inherently uses each subject's own baseline value as the reference point**. The significant "group*time" interaction effect we report directly tests whether the pattern of change over time is different between the groups, already controlling for the starting point.    - Furthermore, as now demonstrated in the revised Table 1, the groups were well-balanced on all measured baseline characteristics (age, gender, BMI, dominance), making it unlikely that these factors are driving the observed effects.   Regarding the control of other potential confounders such as disease duration or comorbidities, we acknowledge this as a valuable point. We have added a statement to the Limitations section to this effect. We agree that future studies with larger sample sizes should prospectively collect and control for these variables using multivariate models. |
| Enhancing the Clinical Argumentation Loop The logical framework of clinical research must form a complete loop: “Data Support → Clinical Validation → Patient Benefit.” “Clinical image comparisons” serve as the core bridge linking ‘data’ to “patient benefit.” The original text only addresses “multi-height data comparisons under standardized conditions,” achieving laboratory-level variable control but failing to provide critical clinical support through actual images: • Supplement with pre- and post-intervention joint imaging (e.g., X-ray, MRI) to visually demonstrate structural benefits like joint space widening and osteophyte reduction. • Incorporate functional assessment images (e.g., gait analysis diagrams, pain VAS trend charts) to demonstrate how data differences translate into tangible clinical outcomes like “reduced joint pain” and “enhanced walking ability.” Without such visual validation, the conclusion that “LWI studies show superiority” lacks the critical link between laboratory data and real patient benefits, failing to form a complete clinical evidence loop. | We thank the reviewer for this profound comment, which elevate the clinical narrative and impact of our work. We agree entirely that having a direct, visual link between biomechanical data and patient benefit is the cornerstone of impactful clinical research.  We are pleased to inform the reviewer that we have directly addressed the core of this feedback through a major revision:  1. Inclusion of Patient-Reported Outcomes (Figure 4): In direct response to Reviewer #1's similar concern, we have now integrated the full results from the Western Ontario and McMaster Universities Osteoarthritis Index (WOMAC) throughout the manuscript. In our initial submission, we focused exclusively on the radiographic outcomes (FTA and MJSW) to maintain a clear and concise narrative on the structural and biomechanical effects of different LWI heights. However, we did indeed collect PROs throughout the study using the Western Ontario and McMaster Universities Osteoarthritis Index (WOMAC). The data show significant improvements in total score and, most notably, the Pain sub-scale in the case group. We believe this inclusion is the most critical step in "closing the clinical loop," as the WOMAC score is a direct, validated measure of the patient's own experience of their symptoms and functional limitations.  2. Regarding the suggestion to include pre- and post-intervention X-rays, we agree that visual evidence is powerful. However, we wish to note that the changes in Medial Joint Space Width (MJSW), while statistically significant and clinically important in the context of disease progression, are subtle at the individual level. These changes are quantifiable by software but are often invisible to the human eye on a standard printed radiograph, and their presentation might therefore be misleading or unconvincing. Therefore, to address the reviewer's request for a "bridge" between data and benefit, we have taken a more strategic and impactful approach:  We have created a new composite figure (Figure 6) that visually constructs the "clinical argumentation loop." This figure integrates our key radiographic endpoint (MJSW) with our patient-reported outcome (WOMAC Pain) data. It visually demonstrates the parallel trends of structural preservation (MJSW) and symptomatic improvement (WOMAC Pain) across the intervention period, thereby creating a direct link between the laboratory data and the patient benefit.  We are confident that these revisions, the inclusion of the WOMAC data and the new synthesizing figure, powerfully close the clinical evidence loop and greatly enhance the translational message of our paper. |
