# Supplementary material for: The impact of insole wedge height on knee alignment, joint space width, and patient-reported outcomes in medial knee OA
Source: PLoS One. 2026 Aug 25;21(8):e0354335. doi: 10.1371/journal.pone.0354335 (PMC13505888; doi:10.1371/journal.pone.0354335)
Supplement: Supplementary file 3 [file pone.0354335.s006.docx]

Revision 3

| Comment | Response |
| --- | --- |
| Reviewer 1 | |
| Reviewer #1: Critical discrepancy between study design and reporting: The abstract and title describe a randomized controlled trial with a case group and a control group, yet the Methods clearly indicate that the case group received four different insole heights sequentially (4, 8, 12, and 16 mm) over time, which constitutes a within-subjects repeated-measures design—not a parallel-group RCT. Could you please clarify whether the control group underwent the same repeated follow-up schedule without any insole, and if so, how the “group×time” interaction can be interpreted as a true between-group comparison when the case group’s time points represent different interventions rather than a single, constant treatment? | We sincerely thank the reviewer for this critical observation. The reviewer is entirely correct. We acknowledge that our design is not a conventional parallel-group RCT where both groups receive a single, fixed intervention across the entire follow-up period.  To clarify: Yes, the control group underwent the exact same repeated follow-up schedule (at baseline, 4 weeks, 8 weeks, and 12 weeks) without any insole intervention to serve as a natural history comparator for disease progression.  We have now substantially revised the description of our design throughout the manuscript to accurately reflect its true nature: a combined design featuring (1) a parallel-group, untreated control arm to track natural history, and (2) a sequential, within-subject dose-escalation intervention arm (testing 4mm, 8mm, and 12mm LWIs) to evaluate the dose-response relationship.  Regarding the interpretation of the "group×time" interaction: We agree with the reviewer that the "time" factor is confounded with "intervention dose" in the case group. Therefore, we do not interpret this interaction as a simple chronological time effect. Instead, we interpret it as evidence that the dose-dependent trajectory of changes observed in the case group (i.e., progressive biomechanical and symptomatic improvement as the wedge height increased from 4mm to 12mm) deviates significantly from the trajectory of natural progression observed in the control group. To provide a robust parallel-group comparison, we have now added explicit supplementary analyses comparing the control group directly to the case group at the final 12mm endpoint (which acts as a true between-group comparison at a single time point), further confirming the superiority of the active intervention over standard care.  **We have revised the Abstract, Methods, discussion, statistical Analysis, and limitations sections below to eliminate this ambiguity and ensure readers clearly understand the unique value of this hybrid design.** |
| Interpretation of side-specific effects: The significant FTA improvement was observed only on the left (non-dominant) side, not on the right (dominant) side. Given that the majority of participants were right-leg dominant, the authors hypothesize that chronic loading on the dominant limb may have led to more “fixed” degenerative changes. If the insole is biomechanically effective, why would it not produce at least a measurable kinematic shift on the dominant side within the 4-week period? Does this asymmetry not undermine the generalizability of the conclusion that “12mm LWI significantly reduces varus alignment,” when this effect appears to be side-dependent and only statistically detectable in the non-dominant limb? | We thank the reviewer for this insightful and critical observation. We agree that the side-dependent nature of the FTA improvement requires careful interpretation and honest acknowledgment.  **1. Why would the insole not produce a measurable kinematic shift on the dominant side within 4 weeks?**  We propose that the **dominant limb's degenerative changes are more structurally advanced** and potentially less amenable to acute biomechanical correction, even though radiographic grading (Kellgren-Lawrence) was matched between limbs. The key distinction is between radiographic severity and structural responsiveness:   - **Radiographic matching** (Kellgren-Lawrence grade <3) ensured both limbs had comparable structural damage at baseline. - **However**, limb dominance is associated with **asymmetric loading history** over decades. The dominant limb experiences higher impact forces, greater propulsive demands, and more cumulative mechanical stress during gait and weight-bearing activities [Turkiewicz et al., 2015; Sadeghi et al., 2000]. - This chronic, elevated loading may lead to **subtle but functionally significant differences** in cartilage composition, subchondral bone stiffness, and periarticular soft tissue adaptation—changes not fully captured by Kellgren-Lawrence grading alone. - These adaptations could render the dominant limb **biomechanically less responsive** to acute correction. The LWI works by inducing foot pronation, which alters the kinematic chain up to the knee. If the dominant knee has more structurally fixed varus due to long-term asymmetric loading, the kinematic correction may be partially dampened before producing a measurable FTA shift within 4 weeks.   In other words: **The absence of a measurable shift on the dominant side does not mean the insole is ineffective; it may simply indicate that the dominant joint requires a longer intervention period or a more substantial mechanical stimulus to produce detectable structural change.**  **2. Does this asymmetry undermine the generalizability of the conclusion?**  **Yes and no. We now phrase our conclusion more precisely:**   - **Yes:** We cannot generalize that "12mm LWI significantly reduces varus alignment" as a universal statement for all limbs. - **No:** We can conclude that **in the non-dominant limb (which represents the joint less exposed to chronic asymmetric overloading) the 12mm LWI produces significant varus correction.** This is clinically meaningful because:   - The non-dominant limb still bears significant weight during gait and is susceptible to OA progression.   - The finding suggests that **earlier intervention** (before asymmetric loading has caused more "fixed" changes) may be more effective.   - It highlights that **limb dominance is a clinically relevant factor** that should be considered when prescribing LWIs and when designing future trials.   **We have revised our conclusion to state:**  "In this cohort, the 12mm LWI significantly reduced varus alignment in the non-dominant limb. The absence of a similar effect in the dominant limb suggests that limb-specific loading history may modulate biomechanical responsiveness, and that the generalizability of this finding is primarily applicable to joints without advanced asymmetric degenerative adaptation. This observation underscores the need for personalized prescription and further investigation into the role of limb dominance in orthotic efficacy."  **3. Does this affect the clinical take-home message?**  No. The clinical significance of our findings is supported by:   - **Consistent improvements in patient-reported outcomes** (WOMAC pain and total score) that were bilateral (not side-specific), indicating that symptom relief is not dependent on FTA correction alone. - **Preserved MJSW bilaterally** in the case group versus narrowing in controls; this structural preservation was seen on both sides, suggesting a disease-modifying effect independent of measurable FTA change. - The **dose-response relationship** (4mm → 8mm → 12mm) showing progressive improvement in the case group, which was not observed in controls.   Thus, while the FTA effect is side-specific, the overall therapeutic benefit of the 12mm LWI is supported by multiple converging lines of evidence. |
| Exclusion of the 16mm data and optimal height claim: The 16mm condition was discontinued due to poor tolerability (11/17 participants) and excluded from analysis. How can the conclusion that “12mm is particularly effective” be justified when the higher dose (16mm) was not fully tested? Was any attempt made to analyze the available data from those who did tolerate the 16mm insole, and how do the authors account for the potential selection bias introduced by excluding nearly two-thirds of the case group at that height? | We thank the reviewer for this important methodological critique. We acknowledge that the 16mm condition was not fully tested and that our conclusion requires more precise wording.  1. How can the conclusion that "12mm is particularly effective" be justified when the higher dose was not fully tested?  We have revised our conclusion to be more precise. We do not claim that 12mm is the *biomechanically optimal* height—we cannot know this because 16mm was not fully tested. Instead, we conclude that 12mm is the most effective height among those that were clinically tolerable and feasible for sustained use.  In other words: 12mm represents the best balance between biomechanical efficacy and patient tolerability within the tested range. This is a clinically meaningful conclusion because an intervention that patients cannot tolerate has zero real-world effectiveness, regardless of its theoretical biomechanical potential.  2. Was any attempt made to analyze the available data from those who did tolerate the 16mm insole?  Yes. We performed an exploratory analysis of the 6 participants who tolerated the 16mm LWI. Their data showed a trend toward further FTA improvement compared to 12mm (mean difference = -0.6°, p = 0.18), but this was not statistically significant due to the very small sample size (n=6). We have now added this as supplementary information (see below).  3. How do we account for the potential selection bias?  We acknowledge this limitation explicitly. The 6 participants who tolerated 16mm may represent a subset with different biomechanical characteristics (e.g., greater baseline joint laxity, less severe OA, different foot morphology, or higher pain tolerance). Therefore, any findings from the 16mm condition are not generalizable to the broader patient population. We have added this to our limitations section.  However, the high dropout rate itself is a clinically significant finding: 11 out of 17 participants could not tolerate the 16mm LWI. This is not a failure of the study—it is a clinically relevant result that informs prescription practice. It demonstrates that 16mm is impractical for routine clinical use, regardless of its theoretical efficacy.  **We have amended the article to account for this comment.** |
| Sample size and power: With an initial recruitment of 44 and a final sample of 34, the authors acknowledge limited power. Could you provide a post-hoc power analysis for the primary outcome (FTA/MJSW) to confirm whether the null findings on the dominant side and for stiffness/physical function subscales are indeed due to a lack of effect or insufficient sample size? | We thank the reviewer for this important methodological question. We have now performed post-hoc power analyses for our primary outcomes to clarify the interpretation of our null findings. 1. Post-hoc power analysis for primary outcomes (FTA and MJSW): Based on our observed effect sizes, the achieved power for the primary analyses was as follows: **Outcome** \| **Effect Size (partial η²)** \| **Observed Power**; FTA (left side) – group×time interaction \| 0.053 \| 0.82; FTA (right side) – group×time interaction \| 0.001 \| 0.06; MJSW (left side) – group×time interaction \| 0.032 \| 0.61; MJSW (right side) – group×time interaction \| 0.009 \| 0.15 These analyses confirm that: - The significant FTA effect on the left (non-dominant) side was adequately powered (observed power = 0.82), supporting the validity of this finding. - The null FTA finding on the right (dominant) side is associated with very low observed power (0.06), meaning we cannot confidently conclude that no effect exists. The absence of a significant effect on the dominant side may indeed reflect insufficient power to detect a smaller effect size, rather than a true lack of biomechanical efficacy. - The MJSW findings (significant interaction on the left side, null on the right side) showed moderate power (0.61) on the left and very low power (0.15) on the right, suggesting the right-side null finding may also be influenced by limited sample size. - For the WOMAC stiffness and physical function subscales, which showed no significant interaction effects, the observed power ranged from 0.28 to 0.42, indicating insufficient power to detect small-to-moderate effects. This suggests that the null findings for these subscales may reflect inadequate sample size rather than a true absence of treatment benefit. 2. Are the null findings due to lack of effect or insufficient sample size? Based on the post-hoc power analysis, we cannot definitively distinguish between true absence of effect and insufficient power for several outcomes. Specifically: - Dominant side FTA: Likely underpowered; the null finding should not be interpreted as evidence that LWI has no effect on the dominant limb. - Stiffness and physical function subscales: Underpowered; these null findings should be interpreted cautiously. - Dominant side MJSW: Underpowered; the null finding is inconclusive. We have revised our manuscript to reflect this interpretation. We now state that the null findings on the dominant side and for certain WOMAC subscales may be attributable to limited statistical power, and we explicitly recommend larger sample sizes in future studies. 3. Should we include the post-hoc power analysis in the manuscript? Yes. We have added a brief summary of the post-hoc power analysis to the Statistical Analysis section and a more detailed presentation in the Limitations section. **We have amended the results, discussion, conclusion, and limitation to account for this comment.** |
| Long-term relevance of radiographic outcomes: While the 4-week intervention shows stabilization of MJSW in the case group versus narrowing in controls, do the authors plan to conduct a longer-term follow-up (e.g., 6–12 months) to assess whether this structural preservation translates into sustained clinical benefit and delayed disease progression, especially given that the WOMAC improvements were also observed? | We thank the reviewer for this important question regarding the long-term clinical relevance of our findings.  **1. Do we plan to conduct a longer-term follow-up?**  We fully agree that long-term follow-up is essential to determine whether the observed structural preservation translates into sustained clinical benefit and delayed disease progression. While our current study was not designed or funded for extended follow-up, we are actively planning a larger prospective cohort study with 12-month follow-up to address this question. In that study, we aim to include dynamic gait analysis and MRI-based cartilage assessment to provide more comprehensive evidence.  **2. Does the absence of long-term follow-up undermine the current findings?**  No. We believe the current findings are clinically meaningful for the following reasons:   - **The 4-week intervention period is consistent with previous LWI studies** that have demonstrated biomechanical and symptomatic effects within this timeframe [Kakihana et al., 2005; Toda et al., 2004; Hinman et al., 2012]. This duration is sufficient to detect acute structural and symptomatic responses. - **The stabilization of MJSW in the case group versus progressive narrowing in the control group** within 4 weeks is clinically significant because it suggests that the LWI may decelerate the natural progression of joint space narrowing—a key radiographic marker of OA progression [Reijman et al., 2005]. - **The concordant improvements in WOMAC scores** (pain and total) provide concurrent patient-reported evidence that the structural preservation is accompanied by tangible clinical benefit.   **However**, we acknowledge that the 4-week timeframe does not allow us to conclude whether these effects are sustained beyond the intervention period. We have revised the manuscript to clearly state this limitation and to emphasize the need for long-term follow-up studies.  **We have, thus, amended the discussion, conclusion, and limitation to account for this comment.** |
| Reviewer 4 | |
| I congratulate you on this study and work . Hope you can come ahead with a larger cohort as well in the near future. | We sincerely thank the reviewer for their kind and encouraging words. We are truly grateful for their positive assessment of our work. We completely agree that larger cohort studies are needed to confirm and extend our findings. As noted in our conclusion, we are actively planning future studies with larger, more diverse samples and longer follow-up periods to further validate the clinical utility of lateral wedge insoles in medial knee OA management |
| Reviewer 5 | |
| The study addresses a clinically relevant topic and suggests that a 12-mm lateral wedge insole may provide the best balance between biomechanical effectiveness and tolerability. However, important methodological clarifications, stronger statistical reporting, improved transparency, and more cautious interpretation of findings are required before the manuscript can be considered for publication. Expand the discussion of previous literature and explain how the present findings differ from existing evidence on lateral wedge insoles. Discuss the potential impact of BMI, physical activity level, occupation, and medication use as confounding factors. Consider adding a limitations paragraph regarding short follow-up duration and lack of functional outcomes. | We sincerely thank the reviewer for their thoughtful and constructive feedback. We have addressed each point systematically as outlined below.  **1. Expand the discussion of previous literature and explain how the present findings differ from existing evidence on lateral wedge insoles.**  We have expanded the discussion to more clearly differentiate our findings from previous LWI studies. Specifically, we now highlight three key distinctions:   - **Dose-response design:** Unlike previous studies that tested only a single wedge height per patient [30–33], our within-subject sequential design allowed direct comparison of multiple heights under standardized conditions, eliminating patient-specific confounding. - **Disease modification evidence:** While most previous studies focused on symptom relief or biomechanical changes (e.g., EKAM reduction), our radiographic findings (stabilized MJSW in the case group versus progressive narrowing in controls) provide preliminary evidence of potential disease-modifying effects. - **Clinical practicality:** Previous studies often did not report tolerability data or dropout rates. Our finding that 64.7% of participants could not tolerate the 16mm wedge provides clinically actionable evidence for prescription practice.   **2. Discuss the potential impact of BMI, physical activity level, occupation, and medication use as confounding factors.**  We acknowledge that these factors were not controlled for in the current study. We have now added a discussion of these potential confounders in the limitations section. Specifically:   - **BMI:** While baseline BMI was not significantly different between groups, higher BMI is associated with greater joint loading and faster OA progression, which may influence responsiveness to LWI. - **Physical activity and occupation:** Activity level and occupational demands affect cumulative joint loading and may influence both disease progression and treatment adherence. We did not systematically assess these factors. - **Medication use:** All participants received routine standard care (pain killers and NSAIDs), but we did not track dosage or frequency. Variability in medication use may have influenced patient-reported outcomes.   **3. Consider adding a limitations paragraph regarding short follow-up duration and lack of functional outcomes.**  We have added a dedicated limitations paragraph addressing:   - **Short follow-up duration:** The 4-week period is insufficient to determine whether the observed structural preservation translates into sustained clinical benefit or delayed disease progression. - **Lack of functional outcomes:** While WOMAC provides patient-reported function, we did not include objective functional measures such as gait analysis, timed walk tests, or balance assessments, which would provide more comprehensive evidence of functional improvement.   Amendments:  **1. Expansion of literature discussion:** We have expanded the discussion to more clearly differentiate our findings from previous LWI studies. We now highlight three key distinctions: (1) our dose-response design allowed direct comparison of multiple heights within the same participants, eliminating patient-specific confounding; (2) our radiographic findings (stabilized MJSW) provide preliminary evidence of potential disease-modifying effects beyond symptom relief; and (3) our systematic documentation of tolerability (64.7% dropout at 16mm) provides clinically actionable evidence for prescription practice.  **2. Confounding factors:** We acknowledge that BMI, physical activity level, occupation, and medication use were not controlled for. While baseline BMI was balanced between groups, we have now added a limitations paragraph explicitly discussing these potential confounders and recommending that future studies account for these variables.  **3. Short follow-up and functional outcomes:** We have added a dedicated limitations paragraph addressing the 4-week follow-up period and the lack of objective functional measures (e.g., gait analysis, timed walk tests). We acknowledge that longer follow-up is needed to determine whether the observed structural preservation translates into sustained clinical benefit.  All revisions have been implemented in the manuscript as described above |
