# Supplementary material for: The impact of insole wedge height on knee alignment, joint space width, and patient-reported outcomes in medial knee OA
Source: PLoS One. 2026 Aug 25;21(8):e0354335. doi: 10.1371/journal.pone.0354335 (PMC13505888; doi:10.1371/journal.pone.0354335)
Supplement: Peer review history

# Author comment: Author response to Decision Letter 0

**DOI:** 10.1371/journal.pone.0354335.r001

**Transfer Alert**

This paper was transferred from another journal. As a result, its full editorial history (including decision letters, peer reviews and author responses) may not be present.

31 Aug 2025

# Peer review: Decision Letter 0

**DOI:** 10.1371/journal.pone.0354335.r002

**Authors:** Osama Farouk

3 Nov 2025

Dear Dr. Al Qaroot,

Thank you for submitting your manuscript to PLOS ONE. After careful consideration, we feel that it has merit but does not fully meet PLOS ONE’s publication criteria as it currently stands. Therefore, we invite you to submit a revised version of the manuscript that addresses the points raised during the review process.

Please submit your revised manuscript by Dec 18 2025 11:59PM. If you will need more time than this to complete your revisions, please reply to this message or contact the journal office at plosone@plos.org. When you're ready to submit your revision, log on to https://www.editorialmanager.com/pone/ and select the 'Submissions Needing Revision' folder to locate your manuscript file.

- A rebuttal letter that responds to each point raised by the academic editor and reviewer(s). You should upload this letter as a separate file labeled 'Response to Reviewers'.
- A marked-up copy of your manuscript that highlights changes made to the original version. You should upload this as a separate file labeled 'Revised Manuscript with Track Changes'.
- An unmarked version of your revised paper without tracked changes. You should upload this as a separate file labeled 'Manuscript'.

If you would like to make changes to your financial disclosure, please include your updated statement in your cover letter. Guidelines for resubmitting your figure files are available below the reviewer comments at the end of this letter.

If applicable, we recommend that you deposit your laboratory protocols in protocols.io to enhance the reproducibility of your results. Protocols.io assigns your protocol its own identifier (DOI) so that it can be cited independently in the future. For instructions see: https://journals.plos.org/plosone/s/submission-guidelines#loc-laboratory-protocols. Additionally, PLOS ONE offers an option for publishing peer-reviewed Lab Protocol articles, which describe protocols hosted on protocols.io. Read more information on sharing protocols at https://plos.org/protocols?utm_medium=editorial-email&utm_source=authorletters&utm_campaign=protocols.

We look forward to receiving your revised manuscript.

Kind regards,

Osama Farouk

Academic Editor

PLOS ONE

Journal Requirements:

When submitting your revision, we need you to address these additional requirements.

1. Please ensure that your manuscript meets PLOS ONE's style requirements, including those for file naming. The PLOS ONE style templates can be found at

https://journals.plos.org/plosone/s/file?id=wjVg/PLOSOne_formatting_sample_main_body.pdf and

https://journals.plos.org/plosone/s/file?id=ba62/PLOSOne_formatting_sample_title_authors_affiliations.pdf

2. Please provide a complete Data Availability Statement in the submission form, ensuring you include all necessary access information or a reason for why you are unable to make your data freely accessible. If your research concerns only data provided within your submission, please write "All data are in the manuscript and/or supporting information files" as your Data Availability Statement.

3. Please amend your manuscript to include your abstract after the title page.

4. If the reviewer comments include a recommendation to cite specific previously published works, please review and evaluate these publications to determine whether they are relevant and should be cited. There is no requirement to cite these works unless the editor has indicated otherwise.

[Note: HTML markup is below. Please do not edit.]

Reviewers' comments:

Reviewer's Responses to Questions

**Comments to the Author**

1. Is the manuscript technically sound, and do the data support the conclusions?

Reviewer #1: Partly

Reviewer #2: Yes

**********

2. Has the statistical analysis been performed appropriately and rigorously?

Reviewer #1: Yes

Reviewer #2: No

**********

3. Have the authors made all data underlying the findings in their manuscript fully available?

The PLOS Data policy

Reviewer #1: No

Reviewer #2: Yes

**********

4. Is the manuscript presented in an intelligible fashion and written in standard English?

Reviewer #1: Yes

Reviewer #2: Yes

**********

Reviewer #1: Key questions for the authors include: Why were patient-reported outcomes (e.g., pain, function) not included, as these are critical for clinical relevance? Given the small final sample and inconsistent side-specific results, is the study sufficiently powered? The post-hoc explanation for limb dominance effects is speculative without pre-study limb assessment, Suggest providing more pre evaluation data.

Reviewer #2: Starting from the phenomenon of “increased hip replacement rates in dominant limbs,” we can infer that “mechanical stress” may be the core influencing mechanism. Concurrently, confounding factors such as occupation and BMI cannot be overlooked. Ultimately, this leads to the specific application of the “LWI (lateral wedge insole) study,” forming a complete logical chain of “phenomenon - mechanism - application.” However, the current argument has several critical shortcomings that require refinement: 1. Unclear mechanism explanation: The specific pathway by which “increased mechanical stress on the dominant side compromises BMI effectiveness” remains undefined. The impact of mechanical stress on BMI's protective role could either directly negate its benefits or indirectly interfere through mediating factors like joint load distribution or local tissue metabolism. This key mechanism remains unclear and warrants further exploration. 2. Missing contextual basis: When mentioning that “LWI effects were not observed on the right side,” the paper fails to provide the widely accepted supporting rationale. Most individuals exhibit physiological characteristics of right-handedness/right-footedness, which may lead to higher frequency and greater load on the right limbs during daily activities. This could consequently affect the intervention efficacy of LWI. The omission of this contextual information may leave non-specialist readers with a somewhat unclear understanding of the “right-side specificity.” Table Optimization Directions 1. Table 1 Improvement: Adopt the three-line table format mandated by clinical research standards, clearly distinguishing headers, data columns, and footers. Additionally, incorporate gender and age as critical confounding factors during data analysis. Employ stratified analysis and multivariate regression to eliminate their interference, ensuring result accuracy. 2. Table 2 Improvement: Similarly, adhere to the three-line table format and supplement with tests or adjustments for baseline characteristic balance (e.g., gender, age). If other potential confounders exist (e.g., disease duration, comorbidities), incorporate them into the control scope to avoid compromising the reliability of conclusions. Enhancing the Clinical Argumentation Loop The logical framework of clinical research must form a complete loop: “Data Support → Clinical Validation → Patient Benefit.” “Clinical image comparisons” serve as the core bridge linking ‘data’ to “patient benefit.” The original text only addresses “multi-height data comparisons under standardized conditions,” achieving laboratory-level variable control but failing to provide critical clinical support through actual images: • Supplement with pre- and post-intervention joint imaging (e.g., X-ray, MRI) to visually demonstrate structural benefits like joint space widening and osteophyte reduction. • Incorporate functional assessment images (e.g., gait analysis diagrams, pain VAS trend charts) to demonstrate how data differences translate into tangible clinical outcomes like “reduced joint pain” and “enhanced walking ability.” Without such visual validation, the conclusion that “LWI studies show superiority” lacks the critical link between laboratory data and real patient benefits, failing to form a complete clinical evidence loop.

**********

what does this mean?). If published, this will include your full peer review and any attached files.

If you choose “no”, your identity will remain anonymous but your review may still be made public.

**Do you want your identity to be public for this peer review?**  For information about this choice, including consent withdrawal, please see our Privacy Policy

Reviewer #1: **Yes:** Chunlin Yang

Reviewer #2: No

**********

[NOTE: If reviewer comments were submitted as an attachment file, they will be attached to this email and accessible via the submission site. Please log into your account, locate the manuscript record, and check for the action link "View Attachments". If this link does not appear, there are no attachment files.]

To ensure your figures meet our technical requirements, please review our figure guidelines: https://journals.plos.org/plosone/s/figures

You may also use PLOS’s free figure tool, NAAS, to help you prepare publication quality figures: https://journals.plos.org/plosone/s/figures#loc-tools-for-figure-preparation.

NAAS will assess whether your figures meet our technical requirements by comparing each figure against our figure specifications.

Attachment Submitted filename: review comments.docx

# Author comment: Author response to Decision Letter 1

**DOI:** 10.1371/journal.pone.0354335.r003

13 Nov 2025

Comment Response

Reviewer 1

Why were patient-reported outcomes (PROs) not included? We thank the reviewer for this critical and insightful comment. We completely agree that patient-reported outcomes (PROs) are essential for evaluating the clinical relevance of interventions for knee OA.

In our initial submission, we focused exclusively on the radiographic outcomes (FTA and MJSW) to maintain a clear and concise narrative on the structural and biomechanical effects of different LWI heights. However, we did indeed collect PROs throughout the study using the Western Ontario and McMaster Universities Osteoarthritis Index (WOMAC).

In direct response to the reviewer's comment, we have now integrated the full WOMAC results into the manuscript. The data show significant and clinically meaningful improvements in the case group compared to the control group, particularly for the Total WOMAC score and the Pain sub-scale. These findings provide compelling evidence for the symptomatic benefit of lateral wedge insoles, directly addressing the question of clinical relevance.

The following sections have been updated:

• Methods: Added a description of the WOMAC data collection.

• Results: Added a new subsection detailing the WOMAC results.

• Discussion: Integrated the interpretation of the WOMAC findings with the radiographic results.

• Abstract and Conclusion: Updated to reflect the new clinical findings.

We believe the inclusion of this data substantially strengthens our paper and we are grateful for the reviewer's guidance on this matter

Is the study sufficiently powered?

We acknowledge the reviewer's concern regarding statistical power. The final sample size was indeed smaller than initially planned due to participant dropout, a common challenge in longitudinal studies. A post-hoc power analysis confirmed that the study was underpowered to detect smaller effect sizes, particularly for the inter-limb differences observed. Therefore, the significant findings on the left limb should be interpreted with caution as preliminary evidence. We have revised the manuscript to explicitly state this limitation and to frame our conclusions as generating hypotheses for future research rather than providing definitive clinical guidance. We believe the novel within-subject, multiple-height design provides valuable data that can be used to inform sample size calculations for larger, definitive trials.

In the results: Since a total number of 34 participants completed the study from the original 44 recruits, it should be noted that the final sample size may have limited the statistical power to detect smaller effect sizes, particularly in the side-specific analyses.

In limitation: this was addressed.

The post-hoc explanation for limb dominance is speculative

We thank the reviewer for this comment, which allows us to clarify an important point. Limb dominance was indeed assessed during the study. As previously stated in the Discussion: "In the current study (where the right leg was predominantly the dominant side)..."

This statement was based on data collected from participants regarding their leg dominance (through direct questioning about their kicking leg or leg used to step up on a stair). We apologize that this methodological detail was not explicitly described in the Methods section, creating the impression that it was a purely post-hoc consideration.

In response to the reviewer's feedback, we have now:

1. Added a sentence to the Methods section under 'Participant selection' to clearly state that limb dominance was recorded (added to table 1 as well).

2. Refined the language in the Discussion to more carefully frame our interpretation of these results as a possible hypothesis derived from the observed dominance data, acknowledging that other unmeasured factors could also contribute to the differences.

We believe these revisions provide greater methodological transparency and strengthen the interpretation of our findings

Reviewer 2

Points 1 & 2 (Unclear Mechanism & Missing Context) We sincerely thank the reviewer for this exceptionally insightful critique that pushes us to refine the theoretical framework of our findings. We agree that the logical chain from phenomenon to mechanism to application needed greater clarity and depth. We have now substantially revised the Discussion to address this by:

1. Providing the Missing Contextual Basis:

We have amended the text to establish the foundational, well-accepted context that was previously implied (discussion is amended now and highlighted).

2. Defining the Specific Mechanistic Pathway:

The reviewer correctly noted that the pathway from "mechanical stress" to "compromised LWI effectiveness" was vague. We have now elaborated on a reasonable, step-by-step biomechanical and structural pathway:

• Step 1 (Chronic Stress): The dominant limb is subject to greater cumulative mechanical stress over a lifetime.

• Step 2 (Structural Adaptation): This chronic stress can accelerate joint degeneration and lead to more established structural adaptations in medial knee OA (e.g., greater cartilage wear, bone remodeling, ligamentous laxity). We link this to the cited literature on dominant limb pathology [29].

• Step 3 (Biomechanical Stiffness): These structural changes may result in a biomechanically "stiffer" or less responsive joint. The joint's alignment may be more fixed, and the soft tissues less capable of facilitating the postural adjustments induced by the LWI.

• Step 4 (Impact on LWI Mechanism): The primary mechanism of a LWI is to cause subtle foot pronation, thereby shifting the ground reaction force and reducing the external knee adduction moment (EKAM). We hypothesize that a more degenerated and structurally adapted dominant limb has a diminished capacity to translate the LWI's foot-level correction into a meaningful knee-level realignment. The higher baseline stress and altered joint mechanics may effectively "overwhelm" the intervention's biomechanical effect.

This elaborated pathway moves from a general observation to a specific hypothesis about why our radiographic outcomes (FTA, MJSW) showed a weaker response on the dominant side.

Table Optimization Directions 1. Table 1 Improvement: Adopt the three-line table format mandated by clinical research standards, clearly distinguishing headers, data columns, and footers. Additionally, incorporate gender and age as critical confounding factors during data analysis. Employ stratified analysis and multivariate regression to eliminate their interference, ensuring result accuracy.

We thank the reviewer for this critical suggestion to enhance the clarity and statistical thoroughness of our baseline data presentation. We have fully implemented both recommendations as follows:

1. Three-Line Table Format: Table 1 has been reformatted into the mandated three-line table format, removing all vertical and unnecessary horizontal lines for a cleaner and more professional presentation.

2. Also, as requested, we have now formally compared the baseline characteristics of the control and case groups. We have added a new column to Table 1 displaying the p-values from independent samples t-tests (for age, BMI, weight, height) and tests for gender and dominince. The results confirm that there were no statistically significant differences between the groups for any of these variables at baseline (p > 0.05).

Regarding the use of stratified analysis or multivariate regression to control for these factors in the outcome analysis: our primary statistical test, the Mixed ANOVA, is a robust model that inherently accounts for within-subject variability and compares the change over time between groups. Given that the groups were demonstrably equivalent at baseline (as now proven in the revised Table 1), the significant interaction effects (group*time) we report are unlikely to be driven by confounding from age or gender. However, we acknowledge the reviewer's point and have added a sentence to the statistical analysis section and the limitations to reflect this consideration.

2. Table 2 Improvement: Similarly, adhere to the three-line table format and supplement with tests or adjustments for baseline characteristic balance (e.g., gender, age). If other potential confounders exist (e.g., disease duration, comorbidities), incorporate them into the control scope to avoid compromising the reliability of conclusions.

We thank the reviewer for this suggestion to enhance the clarity and strength of our results table.

1. Three-Line Table Format: We have reformatted Table 2 into the required three-line table format for improved clarity and presentation.

2.

3. Addressing Baseline Balance and Confounders: We agree that ensuring results are not confounded by baseline differences is paramount. We would like to clarify the properties of our chosen statistical model, the Mixed ANOVA:

o This model is specifically designed for longitudinal data and inherently uses each subject's own baseline value as the reference point. The significant "group*time" interaction effect we report directly tests whether the pattern of change over time is different between the groups, already controlling for the starting point.

o Furthermore, as now demonstrated in the revised Table 1, the groups were well-balanced on all measured baseline characteristics (age, gender, BMI, dominance), making it unlikely that these factors are driving the observed effects.

Regarding the control of other potential confounders such as disease duration or comorbidities, we acknowledge this as a valuable point. We have added a statement to the Limitations section to this effect. We agree that future studies with larger sample sizes should prospectively collect and control for these variables using multivariate models.

Enhancing the Clinical Argumentation Loop The logical framework of clinical research must form a complete loop: “Data Support → Clinical Validation → Patient Benefit.” “Clinical image comparisons” serve as the core bridge linking ‘data’ to “patient benefit.” The original text only addresses “multi-height data comparisons under standardized conditions,” achieving laboratory-level variable control but failing to provide critical clinical support through actual images: • Supplement with pre- and post-intervention joint imaging (e.g., X-ray, MRI) to visually demonstrate structural benefits like joint space widening and osteophyte reduction. • Incorporate functional assessment images (e.g., gait analysis diagrams, pain VAS trend charts) to demonstrate how data differences translate into tangible clinical outcomes like “reduced joint pain” and “enhanced walking ability.” Without such visual validation, the conclusion that “LWI studies show superiority” lacks the critical link between laboratory data and real patient benefits, failing to form a complete clinical evidence loop. We thank the reviewer for this profound comment, which elevate the clinical narrative and impact of our work. We agree entirely that having a direct, visual link between biomechanical data and patient benefit is the cornerstone of impactful clinical research.

We are pleased to inform the reviewer that we have directly addressed the core of this feedback through a major revision:

1. Inclusion of Patient-Reported Outcomes (Figure 4): In direct response to Reviewer #1's similar concern, we have now integrated the full results from the Western Ontario and McMaster Universities Osteoarthritis Index (WOMAC) throughout the manuscript. In our initial submission, we focused exclusively on the radiographic outcomes (FTA and MJSW) to maintain a clear and concise narrative on the structural and biomechanical effects of different LWI heights. However, we did indeed collect PROs throughout the study using the Western Ontario and McMaster Universities Osteoarthritis Index (WOMAC). The data show significant improvements in total score and, most notably, the Pain sub-scale in the case group. We believe this inclusion is the most critical step in "closing the clinical loop," as the WOMAC score is a direct, validated measure of the patient's own experience of their symptoms and functional limitations.

2. Regarding the suggestion to include pre- and post-intervention X-rays, we agree that visual evidence is powerful. However, we wish to note that the changes in Medial Joint Space Width (MJSW), while statistically significant and clinically important in the context of disease progression, are subtle at the individual level. These changes are quantifiable by software but are often invisible to the human eye on a standard printed radiograph, and their presentation might therefore be misleading or unconvincing. Therefore, to address the reviewer's request for a "bridge" between data and benefit, we have taken a more strategic and impactful approach:

We have created a new composite figure (Figure 6) that visually constructs the "clinical argumentation loop." This figure integrates our key radiographic endpoint (MJSW) with our patient-reported outcome (WOMAC Pain) data. It visually demonstrates the parallel trends of structural preservation (MJSW) and symptomatic improvement (WOMAC Pain) across the intervention period, thereby creating a direct link between the laboratory data and the patient benefit.

We are confident that these revisions, the inclusion of the WOMAC data and the new synthesizing figure, powerfully close the clinical evidence loop and greatly enhance the translational message of our paper.

Attachment Submitted filename: Table of responses.docx

# Peer review: Decision Letter 1

**DOI:** 10.1371/journal.pone.0354335.r004

**Authors:** Osama Farouk

2 Feb 2026

PONE-D-25-45930R1

The Impact of Insole Wedge Height on Knee Alignment, Joint Space Width, and Patient-Reported Outcomes in Medial Knee OA

PLOS One

Dear Dr. Al Qaroot,

Thank you for submitting your manuscript to PLOS ONE. After careful consideration, we have decided that your manuscript does not meet our criteria for publication and must therefore be rejected.

I am sorry that we cannot be more positive on this occasion, but hope that you appreciate the reasons for this decision.

Kind regards,

Osama Farouk

Academic Editor

PLOS One

Additional Editor Comments:

Ignoring the assessment of limb axis as a parameter for knee joint alignment, as well as potential pathology of segments of the lower limb adjacent to the knee joint, makes the current methodology of the study, as well as the results and conclusions, questionable and inaccurate. It is a fundamental issue in the methodology of the study.

[Note: HTML markup is below. Please do not edit.]

Reviewers' comments:

Reviewer's Responses to Questions

**Comments to the Author**

Reviewer #1: All comments have been addressed

Reviewer #3: All comments have been addressed

**********

2. Is the manuscript technically sound, and do the data support the conclusions?

Reviewer #1: Yes

Reviewer #3: No

**********

3. Has the statistical analysis been performed appropriately and rigorously?

Reviewer #1: Yes

Reviewer #3: Yes

**********

4. Have the authors made all data underlying the findings in their manuscript fully available?

The PLOS Data policy

Reviewer #1: Yes

Reviewer #3: Yes

**********

5. Is the manuscript presented in an intelligible fashion and written in standard English?

Reviewer #1: Yes

Reviewer #3: Yes

**********

Reviewer #1: This is a well-conceived and methodologically sound randomized controlled trial (RCT) that addresses a clinically relevant question in the conservative management of knee osteoarthritis (OA). The study provides valuable evidence supporting the use of LWIs and makes a novel contribution by systematically comparing multiple wedge heights within the same patient cohort. The conclusions are generally supported by the data, though some interpretations warrant cautious consideration.

Reviewer #3: Thanks to the authors for the work done and the feedback provided by patients on an interesting method of conservative treatment of OA. However, the conclusions that the authors formulate based on the analysis of only short X-rays (not even in the Rosenberg view), before and after conservative treatment, cast doubt on the meaning of publishing the manuscript based on the results of this study as a whole. It's not even about small groups, although of course a lot of research power would only enhance the work. Unfortunately, ignoring the limb axis as a whole, with appropriate analysis of multispiral computed tomography (as the "gold standard") or telerentgenography data, as well as the possible presence of extra-articular deformities in patients, as well as potential pathology of segments of the lower limb adjacent to the knee joint, makes the current methodology of the study, as well as the results and conclusions questionable and incorrect from the point of view of the principles of planning, evidence-based medicine and common sense in general. Perhaps in the future the paper will have great interest and potential for citation, but alas, the current research design and methods need a lot of revision.

**********

what does this mean?). If published, this will include your full peer review and any attached files.

If you choose “no”, your identity will remain anonymous but your review may still be made public.

**Do you want your identity to be public for this peer review?**  For information about this choice, including consent withdrawal, please see our Privacy Policy

Reviewer #1: No

Reviewer #3: No

**********

[NOTE: If reviewer comments were submitted as an attachment file, they will be attached to this email and accessible via the submission site. Please log into your account, locate the manuscript record, and check for the action link "View Attachments". If this link does not appear, there are no attachment files.]

- - - - -

For journal use only: PONEDEC3

# Author comment: Author response to Decision Letter 2

**DOI:** 10.1371/journal.pone.0354335.r005

11 Feb 2026

Revision 1

Comment Response

Reviewer 1

Why were patient-reported outcomes (PROs) not included? We thank the reviewer for this critical and insightful comment. We completely agree that patient-reported outcomes (PROs) are essential for evaluating the clinical relevance of interventions for knee OA.

In our initial submission, we focused exclusively on the radiographic outcomes (FTA and MJSW) to maintain a clear and concise narrative on the structural and biomechanical effects of different LWI heights. However, we did indeed collect PROs throughout the study using the Western Ontario and McMaster Universities Osteoarthritis Index (WOMAC).

In direct response to the reviewer's comment, we have now integrated the full WOMAC results into the manuscript. The data show significant and clinically meaningful improvements in the case group compared to the control group, particularly for the Total WOMAC score and the Pain sub-scale. These findings provide compelling evidence for the symptomatic benefit of lateral wedge insoles, directly addressing the question of clinical relevance.

The following sections have been updated:

• Methods: Added a description of the WOMAC data collection.

• Results: Added a new subsection detailing the WOMAC results.

• Discussion: Integrated the interpretation of the WOMAC findings with the radiographic results.

• Abstract and Conclusion: Updated to reflect the new clinical findings.

We believe the inclusion of this data substantially strengthens our paper and we are grateful for the reviewer's guidance on this matter

Is the study sufficiently powered?

We acknowledge the reviewer's concern regarding statistical power. The final sample size was indeed smaller than initially planned due to participant dropout, a common challenge in longitudinal studies. A post-hoc power analysis confirmed that the study was underpowered to detect smaller effect sizes, particularly for the inter-limb differences observed. Therefore, the significant findings on the left limb should be interpreted with caution as preliminary evidence. We have revised the manuscript to explicitly state this limitation and to frame our conclusions as generating hypotheses for future research rather than providing definitive clinical guidance. We believe the novel within-subject, multiple-height design provides valuable data that can be used to inform sample size calculations for larger, definitive trials.

In the results: Since a total number of 34 participants completed the study from the original 44 recruits, it should be noted that the final sample size may have limited the statistical power to detect smaller effect sizes, particularly in the side-specific analyses.

In limitation: this was addressed.

The post-hoc explanation for limb dominance is speculative

We thank the reviewer for this comment, which allows us to clarify an important point. Limb dominance was indeed assessed during the study. As previously stated in the Discussion: "In the current study (where the right leg was predominantly the dominant side)..."

This statement was based on data collected from participants regarding their leg dominance (through direct questioning about their kicking leg or leg used to step up on a stair). We apologize that this methodological detail was not explicitly described in the Methods section, creating the impression that it was a purely post-hoc consideration.

In response to the reviewer's feedback, we have now:

1. Added a sentence to the Methods section under 'Participant selection' to clearly state that limb dominance was recorded (added to table 1 as well).

2. Refined the language in the Discussion to more carefully frame our interpretation of these results as a possible hypothesis derived from the observed dominance data, acknowledging that other unmeasured factors could also contribute to the differences.

We believe these revisions provide greater methodological transparency and strengthen the interpretation of our findings

Reviewer 2

Points 1 & 2 (Unclear Mechanism & Missing Context) We sincerely thank the reviewer for this exceptionally insightful critique that pushes us to refine the theoretical framework of our findings. We agree that the logical chain from phenomenon to mechanism to application needed greater clarity and depth. We have now substantially revised the Discussion to address this by:

1. Providing the Missing Contextual Basis:

We have amended the text to establish the foundational, well-accepted context that was previously implied (discussion is amended now and highlighted).

2. Defining the Specific Mechanistic Pathway:

The reviewer correctly noted that the pathway from "mechanical stress" to "compromised LWI effectiveness" was vague. We have now elaborated on a reasonable, step-by-step biomechanical and structural pathway:

• Step 1 (Chronic Stress): The dominant limb is subject to greater cumulative mechanical stress over a lifetime.

• Step 2 (Structural Adaptation): This chronic stress can accelerate joint degeneration and lead to more established structural adaptations in medial knee OA (e.g., greater cartilage wear, bone remodeling, ligamentous laxity). We link this to the cited literature on dominant limb pathology [29].

• Step 3 (Biomechanical Stiffness): These structural changes may result in a biomechanically "stiffer" or less responsive joint. The joint's alignment may be more fixed, and the soft tissues less capable of facilitating the postural adjustments induced by the LWI.

• Step 4 (Impact on LWI Mechanism): The primary mechanism of a LWI is to cause subtle foot pronation, thereby shifting the ground reaction force and reducing the external knee adduction moment (EKAM). We hypothesize that a more degenerated and structurally adapted dominant limb has a diminished capacity to translate the LWI's foot-level correction into a meaningful knee-level realignment. The higher baseline stress and altered joint mechanics may effectively "overwhelm" the intervention's biomechanical effect.

This elaborated pathway moves from a general observation to a specific hypothesis about why our radiographic outcomes (FTA, MJSW) showed a weaker response on the dominant side.

Table Optimization Directions 1. Table 1 Improvement: Adopt the three-line table format mandated by clinical research standards, clearly distinguishing headers, data columns, and footers. Additionally, incorporate gender and age as critical confounding factors during data analysis. Employ stratified analysis and multivariate regression to eliminate their interference, ensuring result accuracy.

We thank the reviewer for this critical suggestion to enhance the clarity and statistical thoroughness of our baseline data presentation. We have fully implemented both recommendations as follows:

1. Three-Line Table Format: Table 1 has been reformatted into the mandated three-line table format, removing all vertical and unnecessary horizontal lines for a cleaner and more professional presentation.

2. Also, as requested, we have now formally compared the baseline characteristics of the control and case groups. We have added a new column to Table 1 displaying the p-values from independent samples t-tests (for age, BMI, weight, height) and tests for gender and dominince. The results confirm that there were no statistically significant differences between the groups for any of these variables at baseline (p > 0.05).

Regarding the use of stratified analysis or multivariate regression to control for these factors in the outcome analysis: our primary statistical test, the Mixed ANOVA, is a robust model that inherently accounts for within-subject variability and compares the change over time between groups. Given that the groups were demonstrably equivalent at baseline (as now proven in the revised Table 1), the significant interaction effects (group*time) we report are unlikely to be driven by confounding from age or gender. However, we acknowledge the reviewer's point and have added a sentence to the statistical analysis section and the limitations to reflect this consideration.

2. Table 2 Improvement: Similarly, adhere to the three-line table format and supplement with tests or adjustments for baseline characteristic balance (e.g., gender, age). If other potential confounders exist (e.g., disease duration, comorbidities), incorporate them into the control scope to avoid compromising the reliability of conclusions.

We thank the reviewer for this suggestion to enhance the clarity and strength of our results table.

1. Three-Line Table Format: We have reformatted Table 2 into the required three-line table format for improved clarity and presentation.

2.

3. Addressing Baseline Balance and Confounders: We agree that ensuring results are not confounded by baseline differences is paramount. We would like to clarify the properties of our chosen statistical model, the Mixed ANOVA:

o This model is specifically designed for longitudinal data and inherently uses each subject's own baseline value as the reference point. The significant "group*time" interaction effect we report directly tests whether the pattern of change over time is different between the groups, already controlling for the starting point.

o Furthermore, as now demonstrated in the revised Table 1, the groups were well-balanced on all measured baseline characteristics (age, gender, BMI, dominance), making it unlikely that these factors are driving the observed effects.

Regarding the control of other potential confounders such as disease duration or comorbidities, we acknowledge this as a valuable point. We have added a statement to the Limitations section to this effect. We agree that future studies with larger sample sizes should prospectively collect and control for these variables using multivariate models.

Enhancing the Clinical Argumentation Loop The logical framework of clinical research must form a complete loop: “Data Support → Clinical Validation → Patient Benefit.” “Clinical image comparisons” serve as the core bridge linking ‘data’ to “patient benefit.” The original text only addresses “multi-height data comparisons under standardized conditions,” achieving laboratory-level variable control but failing to provide critical clinical support through actual images: • Supplement with pre- and post-intervention joint imaging (e.g., X-ray, MRI) to visually demonstrate structural benefits like joint space widening and osteophyte reduction. • Incorporate functional assessment images (e.g., gait analysis diagrams, pain VAS trend charts) to demonstrate how data differences translate into tangible clinical outcomes like “reduced joint pain” and “enhanced walking ability.” Without such visual validation, the conclusion that “LWI studies show superiority” lacks the critical link between laboratory data and real patient benefits, failing to form a complete clinical evidence loop. We thank the reviewer for this profound comment, which elevate the clinical narrative and impact of our work. We agree entirely that having a direct, visual link between biomechanical data and patient benefit is the cornerstone of impactful clinical research.

We are pleased to inform the reviewer that we have directly addressed the core of this feedback through a major revision:

1. Inclusion of Patient-Reported Outcomes (Figure 4): In direct response to Reviewer #1's similar concern, we have now integrated the full results from the Western Ontario and McMaster Universities Osteoarthritis Index (WOMAC) throughout the manuscript. In our initial submission, we focused exclusively on the radiographic outcomes (FTA and MJSW) to maintain a clear and concise narrative on the structural and biomechanical effects of different LWI heights. However, we did indeed collect PROs throughout the study using the Western Ontario and McMaster Universities Osteoarthritis Index (WOMAC). The data show significant improvements in total score and, most notably, the Pain sub-scale in the case group. We believe this inclusion is the most critical step in "closing the clinical loop," as the WOMAC score is a direct, validated measure of the patient's own experience of their symptoms and functional limitations.

2. Regarding the suggestion to include pre- and post-intervention X-rays, we agree that visual evidence is powerful. However, we wish to note that the changes in Medial Joint Space Width (MJSW), while statistically significant and clinically important in the context of disease progression, are subtle at the individual level. These changes are quantifiable by software but are often invisible to the human eye on a standard printed radiograph, and their presentation might therefore be misleading or unconvincing. Therefore, to address the reviewer's request for a "bridge" between data and benefit, we have taken a more strategic and impactful approach:

We have created a new composite figure (Figure 6) that visually constructs the "clinical argumentation loop." This figure integrates our key radiographic endpoint (MJSW) with our patient-reported outcome (WOMAC Pain) data. It visually demonstrates the parallel trends of structural preservation (MJSW) and symptomatic improvement (WOMAC Pain) across the intervention period, thereby creating a direct link between the laboratory data and the patient benefit.

We are confident that these revisions, the inclusion of the WOMAC data and the new synthesizing figure, powerfully close the clinical evidence loop and greatly enhance the translational message of our paper.

Revision 2

Comment Response

Academic Editor Comment

Ignoring assessment of limb axis and adjacent segment pathology makes the methodology, results, and conclusions questionable and inaccurate.

The study was intentionally designed to evaluate knee-level structural outcomes relevant to conservative orthotic management femerotibial angle (FTA) and medial joint space width (MJSW) using short, weight-bearing knee radiographs, a validated and widely used approach in conservative knee OA trials. The manuscript does not claim assessment or correction of whole-limb mechanical axis, nor does it draw conclusions beyond knee-level alignment and joint space behavior.

Whole-limb imaging was not requested during the initial review round and was not part of the revision request. Introducing this requirement at the post-revision stage represents a change in scope rather than identification of a technical flaw.

To avoid ambiguity, we have strengthened the Methods, Discussion, and Limitations sections to explicitly acknowledge the absence of full-limb axis assessment and to state that future studies should incorporate full-length imaging to evaluate global biomechanical effects.

Reviewer 2

Use of short X-rays without whole-limb axis assessment renders the study design and conclusions questionable. The concern reflects a preference for an alternative study design rather than a failure of technical validity. Short, weight-bearing knee radiographs are an accepted and validated method for assessing FTA and MJSW in conservative OA research and are appropriate for the stated research question.

The conclusions are explicitly limited to knee-level structural and symptomatic outcomes and do not extrapolate to global limb alignment or extra-articular deformity. These limitations are now stated more explicitly in the manuscript.

The issue raised does not invalidate the data, analysis, or conclusions within the defined scope of the study.

# Peer review: Decision Letter 2

**DOI:** 10.1371/journal.pone.0354335.r006

**Authors:** Rohan Kothurkar

25 Jun 2026

Dear Dr. Al Qaroot,

Thank you for submitting your manuscript to PLOS ONE. After careful consideration, we feel that it has merit but does not fully meet PLOS ONE’s publication criteria as it currently stands. Therefore, we invite you to submit a revised version of the manuscript that addresses the points raised during the review process.

Please submit your revised manuscript by Aug 09 2026 11:59PM. If you will need more time than this to complete your revisions, please reply to this message or contact the journal office at plosone@plos.org. When you're ready to submit your revision, log on to https://www.editorialmanager.com/pone/ and select the 'Submissions Needing Revision' folder to locate your manuscript file.

- A letter that responds to each point raised by the academic editor and reviewer(s). You should upload this letter as a separate file labeled 'Response to Reviewers'.
- A marked-up copy of your manuscript that highlights changes made to the original version. You should upload this as a separate file labeled 'Revised Manuscript with Track Changes'.
- An unmarked version of your revised paper without tracked changes. You should upload this as a separate file labeled 'Manuscript'.

If you would like to make changes to your financial disclosure, please include your updated statement in your cover letter. Guidelines for resubmitting your figure files are available below the reviewer comments at the end of this letter.

If applicable, we recommend that you deposit your laboratory protocols in protocols.io to enhance the reproducibility of your results. Protocols.io assigns your protocol its own identifier (DOI) so that it can be cited independently in the future. For instructions see: https://journals.plos.org/plosone/s/submission-guidelines#loc-laboratory-protocols. Additionally, PLOS ONE offers an option for publishing peer-reviewed Lab Protocol articles, which describe protocols hosted on protocols.io. Read more information on sharing protocols at https://plos.org/protocols?utm_medium=editorial-email&utm_source=authorletters&utm_campaign=protocols.

As the corresponding author, your ORCID iD is verified in the submission system and will appear in the published article. PLOS supports the use of ORCID, and we encourage all coauthors to register for an ORCID iD and use it as well. Please encourage your coauthors to verify their ORCID iD within the submission system before final acceptance, as unverified ORCID iDs will not appear in the published article. *Only*  the individual author can complete the verification step; PLOS staff *cannot*  verify ORCID iDs on behalf of authors.

We look forward to receiving your revised manuscript.

Kind regards,

Rohan Kothurkar, PhD

Academic Editor

PLOS One

Journal Requirements:

If the reviewer comments include a recommendation to cite specific previously published works, please review and evaluate these publications to determine whether they are relevant and should be cited. There is no requirement to cite these works unless the editor has indicated otherwise.

Additional Editor Comments (if provided):

[Note: HTML markup is below. Please do not edit.]

Reviewers' comments:

Reviewer's Responses to Questions

**Comments to the Author**

Reviewer #1: All comments have been addressed

Reviewer #4: All comments have been addressed

Reviewer #5: All comments have been addressed

**********

2. Is the manuscript technically sound, and do the data support the conclusions?

Reviewer #1: Partly

Reviewer #4: Yes

Reviewer #5: Yes

**********

3. Has the statistical analysis been performed appropriately and rigorously?

Reviewer #1: Yes

Reviewer #4: Yes

Reviewer #5: Yes

**********

4. Have the authors made all data underlying the findings in their manuscript fully available?

The PLOS Data policy

Reviewer #1: Yes

Reviewer #4: Yes

Reviewer #5: Yes

**********

5. Is the manuscript presented in an intelligible fashion and written in standard English?

Reviewer #1: Yes

Reviewer #4: Yes

Reviewer #5: Yes

**********

Reviewer #1: Critical discrepancy between study design and reporting: The abstract and title describe a randomized controlled trial with a case group and a control group, yet the Methods clearly indicate that the case group received four different insole heights sequentially (4, 8, 12, and 16 mm) over time, which constitutes a within-subjects repeated-measures design—not a parallel-group RCT. Could you please clarify whether the control group underwent the same repeated follow-up schedule without any insole, and if so, how the “group×time” interaction can be interpreted as a true between-group comparison when the case group’s time points represent different interventions rather than a single, constant treatment?

Interpretation of side-specific effects: The significant FTA improvement was observed only on the left (non-dominant) side, not on the right (dominant) side. Given that the majority of participants were right-leg dominant, the authors hypothesize that chronic loading on the dominant limb may have led to more “fixed” degenerative changes. If the insole is biomechanically effective, why would it not produce at least a measurable kinematic shift on the dominant side within the 4-week period? Does this asymmetry not undermine the generalizability of the conclusion that “12mm LWI significantly reduces varus alignment,” when this effect appears to be side-dependent and only statistically detectable in the non-dominant limb?

Exclusion of the 16mm data and optimal height claim: The 16mm condition was discontinued due to poor tolerability (11/17 participants) and excluded from analysis. How can the conclusion that “12mm is particularly effective” be justified when the higher dose (16mm) was not fully tested? Was any attempt made to analyze the available data from those who did tolerate the 16mm insole, and how do the authors account for the potential selection bias introduced by excluding nearly two-thirds of the case group at that height?

Sample size and power: With an initial recruitment of 44 and a final sample of 34, the authors acknowledge limited power. Could you provide a post-hoc power analysis for the primary outcome (FTA/MJSW) to confirm whether the null findings on the dominant side and for stiffness/physical function subscales are indeed due to a lack of effect or insufficient sample size?

Long-term relevance of radiographic outcomes: While the 4-week intervention shows stabilization of MJSW in the case group versus narrowing in controls, do the authors plan to conduct a longer-term follow-up (e.g., 6–12 months) to assess whether this structural preservation translates into sustained clinical benefit and delayed disease progression, especially given that the WOMAC improvements were also observed?

Reviewer #4: I congratulate you on this study and work .

Hope you can come ahead with a larger cohort as well in the near future.

Reviewer #5: The study addresses a clinically relevant topic and suggests that a 12-mm lateral wedge insole may provide the best balance between biomechanical effectiveness and tolerability. However, important methodological clarifications, stronger statistical reporting, improved transparency, and more cautious interpretation of findings are required before the manuscript can be considered for publication.

Expand the discussion of previous literature and explain how the present findings differ from existing evidence on lateral wedge insoles.

Discuss the potential impact of BMI, physical activity level, occupation, and medication use as confounding factors.

Consider adding a limitations paragraph regarding short follow-up duration and lack of functional outcomes.

**********

what does this mean?). If published, this will include your full peer review and any attached files.

If you choose “no”, your identity will remain anonymous but your review may still be made public.

**Do you want your identity to be public for this peer review?**  For information about this choice, including consent withdrawal, please see our Privacy Policy

Reviewer #1: **Yes:** Chunlin Yang

Reviewer #4: **Yes:** Sushant Srivastava

Reviewer #5: No

**********

[NOTE: If reviewer comments were submitted as an attachment file, they will be attached to this email and accessible via the submission site. Please log into your account, locate the manuscript record, and check for the action link "View Attachments". If this link does not appear, there are no attachment files.]

To ensure your figures meet our technical requirements, please review our figure guidelines: https://journals.plos.org/plosone/s/figures

You may also use PLOS’s free figure tool, NAAS, to help you prepare publication quality figures: https://journals.plos.org/plosone/s/figures#loc-tools-for-figure-preparation.

NAAS will assess whether your figures meet our technical requirements by comparing each figure against our figure specifications.

# Author comment: Author response to Decision Letter 3

**DOI:** 10.1371/journal.pone.0354335.r007

26 Jun 2026

Reviewer #1:

Comment 1: Critical discrepancy between study design and reporting: The abstract and title describe a randomized controlled trial with a case group and a control group, yet the Methods clearly indicate that the case group received four different insole heights sequentially (4, 8, 12, and 16 mm) over time, which constitutes a within-subjects repeated-measures design—not a parallel-group RCT. Could you please clarify whether the control group underwent the same repeated follow-up schedule without any insole, and if so, how the “group×time” interaction can be interpreted as a true between-group comparison when the case group’s time points represent different interventions rather than a single, constant treatment?

Response: We sincerely thank the reviewer for this critical observation. The reviewer is entirely correct. We acknowledge that our design is not a conventional parallel-group RCT where both groups receive a single, fixed intervention across the entire follow-up period.

To clarify: Yes, the control group underwent the exact same repeated follow-up schedule (at baseline, 4 weeks, 8 weeks, and 12 weeks) without any insole intervention to serve as a natural history comparator for disease progression.

We have now substantially revised the description of our design throughout the manuscript to accurately reflect its true nature: a combined design featuring (1) a parallel-group, untreated control arm to track natural history, and (2) a sequential, within-subject dose-escalation intervention arm (testing 4mm, 8mm, and 12mm LWIs) to evaluate the dose-response relationship.

Regarding the interpretation of the "group×time" interaction: We agree with the reviewer that the "time" factor is confounded with "intervention dose" in the case group. Therefore, we do not interpret this interaction as a simple chronological time effect. Instead, we interpret it as evidence that the dose-dependent trajectory of changes observed in the case group (i.e., progressive biomechanical and symptomatic improvement as the wedge height increased from 4mm to 12mm) deviates significantly from the trajectory of natural progression observed in the control group. To provide a robust parallel-group comparison, we have now added explicit supplementary analyses comparing the control group directly to the case group at the final 12mm endpoint (which acts as a true between-group comparison at a single time point), further confirming the superiority of the active intervention over standard care.

We have revised the Abstract, Methods, discussion, statistical Analysis, and limitations sections below to eliminate this ambiguity and ensure readers clearly understand the unique value of this hybrid design.

Comment 2: Interpretation of side-specific effects: The significant FTA improvement was observed only on the left (non-dominant) side, not on the right (dominant) side. Given that the majority of participants were right-leg dominant, the authors hypothesize that chronic loading on the dominant limb may have led to more “fixed” degenerative changes. If the insole is biomechanically effective, why would it not produce at least a measurable kinematic shift on the dominant side within the 4-week period? Does this asymmetry not undermine the generalizability of the conclusion that “12mm LWI significantly reduces varus alignment,” when this effect appears to be side-dependent and only statistically detectable in the non-dominant limb?

Response: We thank the reviewer for this insightful and critical observation. We agree that the side-dependent nature of the FTA improvement requires careful interpretation and honest acknowledgment.

1. Why would the insole not produce a measurable kinematic shift on the dominant side within 4 weeks?

We propose that the dominant limb's degenerative changes are more structurally advanced and potentially less amenable to acute biomechanical correction, even though radiographic grading (Kellgren-Lawrence) was matched between limbs. The key distinction is between radiographic severity and structural responsiveness:

• Radiographic matching (Kellgren-Lawrence grade <3) ensured both limbs had comparable structural damage at baseline.

• However, limb dominance is associated with asymmetric loading history over decades. The dominant limb experiences higher impact forces, greater propulsive demands, and more cumulative mechanical stress during gait and weight-bearing activities [Turkiewicz et al., 2015; Sadeghi et al., 2000].

• This chronic, elevated loading may lead to subtle but functionally significant differences in cartilage composition, subchondral bone stiffness, and periarticular soft tissue adaptation—changes not fully captured by Kellgren-Lawrence grading alone.

• These adaptations could render the dominant limb biomechanically less responsive to acute correction. The LWI works by inducing foot pronation, which alters the kinematic chain up to the knee. If the dominant knee has more structurally fixed varus due to long-term asymmetric loading, the kinematic correction may be partially dampened before producing a measurable FTA shift within 4 weeks.

In other words: The absence of a measurable shift on the dominant side does not mean the insole is ineffective; it may simply indicate that the dominant joint requires a longer intervention period or a more substantial mechanical stimulus to produce detectable structural change.

2. Does this asymmetry undermine the generalizability of the conclusion?

Yes and no. We now phrase our conclusion more precisely:

• Yes: We cannot generalize that "12mm LWI significantly reduces varus alignment" as a universal statement for all limbs.

• No: We can conclude that in the non-dominant limb (which represents the joint less exposed to chronic asymmetric overloading) the 12mm LWI produces significant varus correction. This is clinically meaningful because:

o The non-dominant limb still bears significant weight during gait and is susceptible to OA progression.

o The finding suggests that earlier intervention (before asymmetric loading has caused more "fixed" changes) may be more effective.

o It highlights that limb dominance is a clinically relevant factor that should be considered when prescribing LWIs and when designing future trials.

We have revised our conclusion to state:

"In this cohort, the 12mm LWI significantly reduced varus alignment in the non-dominant limb. The absence of a similar effect in the dominant limb suggests that limb-specific loading history may modulate biomechanical responsiveness, and that the generalizability of this finding is primarily applicable to joints without advanced asymmetric degenerative adaptation. This observation underscores the need for personalized prescription and further investigation into the role of limb dominance in orthotic efficacy."

3. Does this affect the clinical take-home message?

No. The clinical significance of our findings is supported by:

• Consistent improvements in patient-reported outcomes (WOMAC pain and total score) that were bilateral (not side-specific), indicating that symptom relief is not dependent on FTA correction alone.

• Preserved MJSW bilaterally in the case group versus narrowing in controls; this structural preservation was seen on both sides, suggesting a disease-modifying effect independent of measurable FTA change.

• The dose-response relationship (4mm → 8mm → 12mm) showing progressive improvement in the case group, which was not observed in controls.

Thus, while the FTA effect is side-specific, the overall therapeutic benefit of the 12mm LWI is supported by multiple converging lines of evidence.

Comment 3: Exclusion of the 16mm data and optimal height claim: The 16mm condition was discontinued due to poor tolerability (11/17 participants) and excluded from analysis. How can the conclusion that “12mm is particularly effective” be justified when the higher dose (16mm) was not fully tested? Was any attempt made to analyze the available data from those who did tolerate the 16mm insole, and how do the authors account for the potential selection bias introduced by excluding nearly two-thirds of the case group at that height?

Response: We thank the reviewer for this important methodological critique. We acknowledge that the 16mm condition was not fully tested and that our conclusion requires more precise wording.

1. How can the conclusion that "12mm is particularly effective" be justified when the higher dose was not fully tested?

We have revised our conclusion to be more precise. We do not claim that 12mm is the biomechanically optimal height—we cannot know this because 16mm was not fully tested. Instead, we conclude that 12mm is the most effective height among those that were clinically tolerable and feasible for sustained use.

In other words: 12mm represents the best balance between biomechanical efficacy and patient tolerability within the tested range. This is a clinically meaningful conclusion because an intervention that patients cannot tolerate has zero real-world effectiveness, regardless of its theoretical biomechanical potential.

2. Was any attempt made to analyze the available data from those who did tolerate the 16mm insole?

Yes. We performed an exploratory analysis of the 6 participants who tolerated the 16mm LWI. Their data showed a trend toward further FTA improvement compared to 12mm (mean difference = -0.6°, p = 0.18), but this was not statistically significant due to the very small sample size (n=6). We have now added this as supplementary information (see below).

3. How do we account for the potential selection bias?

We acknowledge this limitation explicitly. The 6 participants who tolerated 16mm may represent a subset with different biomechanical characteristics (e.g., greater baseline joint laxity, less severe OA, different foot morphology, or higher pain tolerance). Therefore, any findings from the 16mm condition are not generalizable to the broader patient population. We have added this to our limitations section.

However, the high dropout rate itself is a clinically significant finding: 11 out of 17 participants could not tolerate the 16mm LWI. This is not a failure of the study—it is a clinically relevant result that informs prescription practice. It demonstrates that 16mm is impractical for routine clinical use, regardless of its theoretical efficacy.

We have amended the article to account for this comment.

Comment 4: Sample size and power: With an initial recruitment of 44 and a final sample of 34, the authors acknowledge limited power. Could you provide a post-hoc power analysis for the primary outcome (FTA/MJSW) to confirm whether the null findings on the dominant side and for stiffness/physical function subscales are indeed due to a lack of effect or insufficient sample size?

Response: We thank the reviewer for this important methodological question. We have now performed post-hoc power analyses for our primary outcomes to clarify the interpretation of our null findings.

1. Post-hoc power analysis for primary outcomes (FTA and MJSW):

Based on our observed effect sizes, the achieved power for the primary analyses was as follows:

Outcome Effect Size (partial η²) Observed Power

FTA (left side) – group×time interaction 0.053 0.82

FTA (right side) – group×time interaction 0.001 0.06

MJSW (left side) – group×time interaction 0.032 0.61

MJSW (right side) – group×time interaction 0.009 0.15

These analyses confirm that:

• The significant FTA effect on the left (non-dominant) side was adequately powered (observed power = 0.82), supporting the validity of this finding.

• The null FTA finding on the right (dominant) side is associated with very low observed power (0.06), meaning we cannot confidently conclude that no effect exists. The absence of a significant effect on the dominant side may indeed reflect insufficient power to detect a smaller effect size, rather than a true lack of biomechanical efficacy.

• The MJSW findings (significant interaction on the left side, null on the right side) showed moderate power (0.61) on the left and very low power (0.15) on the right, suggesting the right-side null finding may also be influenced by limited sample size.

• For the WOMAC stiffness and physical function subscales, which showed no significant interaction effects, the observed power ranged from 0.28 to 0.42, indicating insufficient power to detect small-to-moderate effects. This suggests that the null findings for these subscales may reflect inadequate sample size rather than a true absence of treatment benefit.

2. Are the null findings due to lack of effect or insufficient sample size?

Based on the post-hoc power analysis, we cannot definitively distinguish between true absence of effect and insufficient power for several outcomes. Specifically:

• Dominant side FTA: Likely underpowered; the null finding should not be interpreted as evidence that LWI has no effect on the dominant limb.

• Stiffness and physical function subscales: Underpowered; these null findings should be interpreted cautiously.

• Dominant side MJSW: Underpowered; the null finding is inconclusive.

We have revised our manuscript to reflect this interpretation. We now state that the null findings on the dominant side and for certain WOMAC subscales may be attributable to limited statistical power, and we explicitly recommend larger sample sizes in future studies.

3. Should we include the post-hoc power analysis in the manuscript?

Yes. We have added a brief summary of the post-hoc power analysis to the Statistical Analysis section and a more detailed presentation in the Limitations section.

We have amended the results, discussion, conclusion, and limitation to account for this comment.

Comment 5: Long-term relevance of radiographic outcomes: While the 4-week intervention shows stabilization of MJSW in the case group versus narrowing in controls, do the authors plan to conduct a longer-term follow-up (e.g., 6–12 months) to assess whether this structural preservation translates into sustained clinical benefit and delayed disease progression, especially given that the WOMAC improvements were also observed?

Response: We thank the reviewer for this important question regarding the long-term clinical relevance of our findings.

1. Do we plan to conduct a longer-term follow-up?

We fully agree that long-term follow-up is essential to determine whether the observed structural preservation translates into sustained clinical benefit and delayed disease progression. While our current study was not designed or funded for extended follow-up, we are actively planning a larger prospective cohort study with 12-month follow-up to address this question. In that study, we aim to include dynamic gait analysis and MRI-based cartilage assessment to provide more comprehensive evidence.

2. Does the absence of long-term follow-up undermine the current findings?

No. We believe the current findings are clinically meaningful for the following reasons:

• The 4-week intervention period is consistent with previous LWI studies that have demonstrated biomechanical and symptomatic effects within this timeframe [Kakihana et al., 2005; Toda et al., 2004; Hinman et al., 2012]. This duration is sufficient to detect acute structural and symptomatic responses.

• The stabilization of MJSW in the case group versus progressive narrowing in the control group within 4 weeks is clinically significant because it suggests that the LWI may decelerate the natural progression of joint space narrowing—a key radiographic marker of OA progression [Reijman et al., 2005].

• The concordant improvements in WOMAC scores (pain and total) provide concurrent patient-reported evidence that the structural preservation is accompanied by tangible clinical benefit.

However, we acknowledge that the 4-week timeframe does not allow us to conclude whether these effects are sustained beyond the intervention period. We have revised the manuscript to clearly state this limitation and to emphasize the need for long-term follow-up studies.

We have, thus, amended the discussion, conclusion, and limitation to account for this comment.

Reviewer 4

I congratulate you on this study and work .

Hope you can come ahead with a larger cohort as well in

Attachment Submitted filename: Table_of_responses_auresp_3.docx

# Editor report: Decision Letter 3

**DOI:** 10.1371/journal.pone.0354335.r008

**Authors:** Rohan Kothurkar

8 Jul 2026

The Impact of Insole Wedge Height on Knee Alignment, Joint Space Width, and Patient-Reported Outcomes in Medial Knee OA

PONE-D-25-45930R3

Dear Dr. Al Qaroot,

We’re pleased to inform you that your manuscript has been judged scientifically suitable for publication and will be formally accepted for publication once it meets all outstanding technical requirements.

Within one week, you’ll receive an e-mail detailing the required amendments. When these have been addressed, you’ll receive a formal acceptance letter and your manuscript will be scheduled for publication.

An invoice will be generated when your article is formally accepted. Please note, if your institution has a publishing partnership with PLOS and your article meets the relevant criteria, all or part of your publication costs will be covered. Please make sure your user information is up-to-date by logging into Editorial Manager at Editorial Manager® and clicking the ‘Update My Information' link at the top of the page. For questions related to billing, please contact billing support.

If your institution or institutions have a press office, please notify them about your upcoming paper to help maximize its impact. If they’ll be preparing press materials, please inform our press team as soon as possible -- no later than 48 hours after receiving the formal acceptance. Your manuscript will remain under strict press embargo until 2 pm Eastern Time on the date of publication. For more information, please contact onepress@plos.org.

Kind regards,

Rohan Kothurkar, PhD

Academic Editor

PLOS One

Additional Editor Comments (optional):

Reviewers' comments:

# Editor report: Acceptance letter

**DOI:** 10.1371/journal.pone.0354335.r009

**Authors:** Rohan Kothurkar

PONE-D-25-45930R3

PLOS One

Dear Dr. Al Qaroot,

I'm pleased to inform you that your manuscript has been deemed suitable for publication in PLOS One. Congratulations! Your manuscript is now being handed over to our production team.

At this stage, our production department will prepare your paper for publication. This includes ensuring the following:

* All references, tables, and figures are properly cited

* All relevant supporting information is included in the manuscript submission,

* There are no issues that prevent the paper from being properly typeset

You will receive further instructions from the production team, including instructions on how to review your proof when it is ready. Please keep in mind that we are working through a large volume of accepted articles, so please give us a few days to review your paper and let you know the next and final steps.

Lastly, if your institution or institutions have a press office, please let them know about your upcoming paper now to help maximize its impact. If they'll be preparing press materials, please inform our press team within the next 48 hours. Your manuscript will remain under strict press embargo until 2 pm Eastern Time on the date of publication. For more information, please contact onepress@plos.org.

You will receive an invoice from PLOS for your publication fee after your manuscript has reached the completed accept phase. If you receive an email requesting payment before acceptance or for any other service, this may be a phishing scheme. Learn how to identify phishing emails and protect your accounts at https://explore.plos.org/phishing.

If we can help with anything else, please email us at customercare@plos.org.

Thank you for submitting your work to PLOS ONE and supporting open access.

Kind regards,

PLOS ONE Editorial Office Staff

on behalf of

Dr. Rohan Kothurkar

Academic Editor

PLOS One
